# A Metagenome-Wide Association Study and Arrayed Mutant Library Confirm *Acetobacter* Lipopolysaccharide Genes Are Necessary for Association with *Drosophila melanogaster*

**DOI:** 10.1534/g3.117.300530

**Published:** 2018-02-27

**Authors:** K. Makay White, Melinda K. Matthews, Rachel C. Hughes, Andrew J. Sommer, Joel S. Griffitts, Peter D. Newell, John M. Chaston

**Affiliations:** * Microbiology and Molecular Biology Department, Brigham Young University, Provo, Utah 84602; † Plant and Wildlife Sciences Department, Brigham Young University, Provo Utah 84602; ‡ Department of Biological Sciences, State University of New York at Oswego, New York 13126

**Keywords:** *Acetobacter*, *Drosophila*, lipopolysaccharide, Lipid A, mutant library, Mutant Screen Report

## Abstract

A metagenome wide association (MGWA) study of bacterial host association determinants in *Drosophila* predicted that LPS biosynthesis genes are significantly associated with host colonization. We were unable to create site-directed mutants for each of the predicted genes in *Acetobacter*, so we created an arrayed transposon insertion library using *Acetobacter fabarum* DsW_054 isolated from *Drosophila*. Creation of the *A. fabarum* DsW_054 gene knock-out library was performed by combinatorial mapping and Illumina sequencing of random transposon insertion mutants. Transposon insertion locations for 6,418 mutants were successfully mapped, including hits within 63% of annotated genes in the *A. fabarum* DsW_054 genome. For 45/45 members of the library, insertion sites were verified by arbitrary PCR and Sanger sequencing. Mutants with insertions in four different LPS biosynthesis genes were selected from the library to validate the MGWA predictions. Insertion mutations in two genes biosynthetically upstream of Lipid-A formation, *lpxC* and *lpxB*, show significant differences in host association, whereas mutations in two genes encoding LPS biosynthesis functions downstream of Lipid-A biosynthesis had no effect. These results suggest an impact of bacterial cell surface molecules on the bacterial capacity for host association. Also, the transposon insertion mutant library will be a useful resource for ongoing research on the genetic basis for *Acetobacter* traits.

Animal associated microbes (‘microbiota’) influence numerous phenotypes of their hosts, including metabolic function, mental health and various diseases (De Palma *et al.* 2015; Ussar *et al.* 2015; Cammarota *et al.* 2015; Verdu *et al.* 2015; Bienenstock *et al.* 2015; Petra *et al.* 2015). The taxonomic and functional complexity of the microbiota challenges our ability to define how these microbes associate with and influence traits of their hosts. The *Drosophila melanogaster* microbiota is relatively simple, mainly comprised of yeasts, acetic acid bacteria (AABs) and lactic acid bacteria (LABs), together with less abundant but highly prevalent *Enterobacteriaceae* (Wong *et al.* 2011; Broderick and Lemaitre 2012; Erkosar *et al.* 2013; Ma *et al.* 2015; Cox and Gilmore 2007; Bakula 1969; Brummel *et al.* 2004; Corby-Harris *et al.* 2007), and varies in composition and abundance between and within individual flies, including with age (Blum *et al.* 2013; Broderick *et al.* 2014; Wong *et al.* 2013; Ren *et al.* 2007). This apparent inconstancy is determined by numerous factors, including host genetic selection, environmental sampling, and dietary effects (Blum *et al.* 2013; Chaston *et al.* 2015; Wong *et al.* 2015). For example, the fly microbiota is replenished through the diet, and bacterial loads can be reduced by frequent transfer of flies to sterile diets (Blum *et al.* 2013; Broderick *et al.* 2014). Host genetic influences include reciprocal interactions between the host and the diet since fly diets inoculated with a defined fly microbiota *vs.* the same microbiota and flies, have a different ending microbiota composition (Wong *et al.* 2015). Also, host genotype can influence microbial composition since different fly genotypes reared from birth with the same starting microbiota can have different ending compositions of microbes (Chaston *et al.* 2015; Wong *et al.* 2015). However, under laboratory conditions there is no evidence that the host retains specific bacterial taxa or functions within or across generations, or for a host phylogenetic signal on the identity of associated microbes (Wong *et al.* 2013). The variability in bacterial abundance and composition is an important factor in animal phenotype studies (Clark *et al.* 2015; Vandeputte *et al.* 2017).

Microbial genetic determinants also influence the abundance of bacteria associated with *D. melanogaster*. For example, the typical laboratory fly associated microbiota do not produce sufficient uracil for the *Drosophila* gut to elicit an immune response, allowing the insect to respond differently to normal and pathogenic microbes (Lee *et al.* 2013; Ha *et al.* 2009). Additionally, D-alanylated teichoic acids on the cell walls of *L. plantarum* were sensed by the Drosophila enterocytes, enhancing host digestion and promoting growth and maturation of the host (Matos *et al.* 2017). *Acetobacter* species that degrade uric acid can outcompete those that do not when associated with *Drosophila* (Winans *et al.* 2017). Together, these studies have identified some bacterial genes that influence how bacteria associate with *D. melanogaster*; our goal was to identify new genes with similar influence.

Because there were no successful approaches for site-directed mutagenesis in fruit fly isolates of *Acetobacter*, and our preliminary efforts to develop such approaches failed, we created an arrayed transposon insertion library of *Acetobacter* mutants. Arrayed mutant libraries allow for high-throughput identification of thousands of knock-out mutants at once. One method was described by Goodman *et al.* (Goodman *et al.* 2011; Goodman *et al.* 2009), where transposon insertion mutants were arrayed in 96-well plates, combined into sequencing pools, and sequenced in one sequencing run. The mutants were used to determine bacterial functions necessary for survival in the mouse gut (Goodman *et al.* 2009). Others have screened arrayed transposon insertion libraries of *Klebsiella pneumoniae* for antibiotic sensitivity (Ramage *et al.* 2017); and of *L. plantarum* for bacterial genes that promote host growth (Matos *et al.* 2017). In this study we created an arrayed library of 6,418 *A. fabarum* DsW_054 transposon insertion mutants and report its first use by testing five lipopolysaccharide (LPS) biosynthesis mutants for their ability to associate with *D. melanogaster*.

## Materials and Methods

### Bacterial and fly growth media and conditions

*D. melanogaster* Canton-S flies were grown at 25° on a yeast glucose diet containing 100 g/ liter brewer’s yeast (inactive) (MP Biomedicals), 100 g/ liter glucose (Sigma), 12 g/ liter agar (Apex), and preservatives (0.04% phosphoric acid and 0.42% propionic acid (Sigma)) on a 12-h-light/12-h-dark cycle.

Bacterial strains used in the study are included in Table 1. Media used included lysogeny broth (LB)/agar (Sigma), modified MRS (mMRS) broth/agar and potato dextrose broth/ agar (Sigma). The plasmid bearing *E. coli* strain was cultured with 50 μg/ml kanamycin and *Acetobacter* gene knock-out mutants were cultured with 30 μg/ml chloramphenicol and 50 μg/ml kanamycin. All bacterial strains were cultured at 30°. *A. fabarum* DsW_054 was cultured in potato dextrose broth prior to matings.

**Table 1:** Bacterial strains used in this study

**Strain Name**	**Abbreviation**	**Preferred Medium**	**Citation**
*A. fabarum* DsW_054		mMRS	Winans *et al.* (2017)
*Escherichia coli* S17 pJG714		LB-kan	This study (see Figure S1 in File S1)
*A. fabarum* DsW_054 Tn*5*::lpxC	lpxC	mMRS	This study
*A. fabarum* DsW_054 Tn5::lpxB	lpxB	mMRS	This study
*A. fabarum* DsW_054 Tn5::lpxK	lpxK	mMRS	This study
*A. fabarum* DsW_054 Tn5::gmhD_1	gmhD_1	mMRS	This study
*A. fabarum* DsW_054 Tn5::gmhD_2	gmhD_2	mMRS	This study

### Creation of an arrayed mutant library

An arrayed transposon insertion library was created in two steps: by conjugally transferring the Tn*5* transposon vector pJG714 into *A. fabarum* DsW_054; and by arraying the transposants into 96-well plates. *A. fabarum* DsW_054 cells were cultured in potato medium for 24 -36 hr at 30° to OD_600_ = 0.6 and mixed with 18-24 h cultures of *E. coli* S17-pir (OD_600_ = 1.0) containing the plasmid pJG714, cultured in LB-kanamycin. Prior to mixing, the cells were washed three times in potato medium, 6 ml *A. fabarum* DsW_054 were condensed to 100 μl, and 3 ml *E. coli* were re-suspended in 500 μl. The two strains were mixed in a 1:1 volumetric ratio and transferred to potato medium plates in 50 μl spots. After 4 hr at 30° the cells were collected from the plate in 1ml potato medium, dilution plated (1:50) onto 2X-YPG plates containing kanamycin and chloramphenicol, incubated at 30° for four days, and stored at 4° for no more than 1 week before arraying in 96-well plates. Colonies were individually picked into mMRS broth in 96-well plates to array the transposon insertion library. Each plate was then sealed with Parafilm and incubated with gentle shaking for 48 hr at 30° until most cell densities were between OD_600_ 0.5 and 1.0, and frozen in mMRS-25% glycerol.

### Combinatorial mapping and sequencing

To identify the insertion site of each mutant we employed a combinatorial mapping approach using an Eppendorf EpMotion 5075 TMX pipetting robot. A 24 bit binary barcode was assigned to each well of each 96 well plate, corresponding to the presence (1) or absence (0) of the bacteria from each well in the corresponding pooled vials (Goodman *et al.* 2011). Intermediate sets of 24 pools were created from five 96-well plates at a time in the attached thermocycler feature held at 4° by pipetting 10 μl to each intermediate pool for which a ‘1’ was assigned to that sample of the 96-well plate. The different pools were stored at -20° for a maximum of 2 months. Once all the intermediate pools had been created, a final set of 24 pools was created by mixing all intermediate pools from the same barcode positions (*e.g.*, all ‘Pool 1’ tubes) in equal volumetric ratios. DNA was extracted separately from each of the pools using the DNeasy PowerLyzer Microbial Kit (Qiagen cat# 12255-50).

For Illumina sequencing, each of the 24 final pools was assigned a unique 6 bp indexing barcode that was introduced by PCR (for library preparation details and primer sequences, see File S2). Briefly, DNA was fragmented using a DNA fragmentase, the fragments were C-tailed, and Illumina indexing and sequencing primers (Table S1 in File S1) were added via two rounds of PCR.

### Data analysis

The Illumina sequencing data were mapped to the *A. fabarum* DsW_054 genome using a previously-published TnSeq pipeline (Arnold *et al.* 2017). Afterward, a 24-bit barcode was assigned to each mapped insertion site in the genome using a threshold of 50 reads per sequencing pool. For example, if there were more than 50 reads for a particular insertion site in sequencing pool 1, a 1 was assigned at the first position in the barcode; if there were less than 50 reads in the second sequencing pool, a 0 was assigned at the second barcode position, etc. We used 50 reads as a cutoff point, but most sites had more than 200 reads for each positive indexing primer. Insertion site barcodes were then matched to the barcodes from the original combinatorial mapping assignments for the well location in the 96-well plate library. The complete mapping to the library is summarized in File S3.

### Library validation

Arbitrary PCR was used to validate a subset of the mapped insertion sites for the arrayed library. A transposon specific primer was paired with arbitrary primers for the first round and the second round used a primer specific to the transposon and a primer specific to the tail of the initial arbitrary primers (Table S1 in File S1). A single colony that was suspended in 10 μl H_2_O and boiled for 10 min at 99° served as the template. For first round arbitrary PCR we mixed 14 μl H_2_O, 2.5 μl 10x (NH_4_)_2_SO_4_ buffer, 1 μl DMSO, 2 μl MgCl_2_ (25 mM), 1 ul dNTP (10 μM), 1 μl Arb1 primer (20 μM), 1 μl Arb6 primer (20 μM), 0.5 μl 133 primer (20 μM), 1 μl Taq polymerase 1U/μl (Thermo Scientific), and 1 μl template (boiled cell preparation). We ran the reaction using the following program: 94° for 3:00 followed by six cycles of 94° for 0:20, 30° for 0:20 and 72° for 1:00, followed by 30 cycles of 94° for 0:20, 45° for 0:20, 72° for 1:00 and finished at 72° for 5:00.

This was followed by a second round of arbitrary PCR. For each sample we mixed 31.25 μl H_2_O, 5 μl 10x (NH_4_)_2_SO_4_ buffer, 2 μl DMSO, 4 μl MgCl_2_ (25mM), 2 μl dNTP (10 μM), 2 μl Arb2 primer (20 μM), 2 μl 134 primer (20 μM), 1 μl Taq polymerase 1U/μl (Thermo Scientific, 1 μl Template (Product from first round). We ran the solution at 94° for 1:00 followed by 35 cycles of 94° for 0:15, 52° for 0:20, 72° for 1:00, and finish at 72° for 5:00. The 150-700 bp product was visualized on a 1% agarose gel to verify amplification before sequencing via Sanger sequencing. The specific insertion site was manually mapped to the *A. fabarum* DsW_054 genome using the BLAST feature in RAST (Wattam *et al.* 2017).

### Metagenome-wide-association study

A metagenome wide association study (MGWA) was performed to predict bacterial genes necessary to associate with *D. melanogaster* using previously published phenotype and genotype data (Chaston *et al.* 2014). Briefly, in the previous study CFU abundances were collected from pooled homogenates of five whole female flies that had been individually reared with one of 41 different, genome-sequenced bacterial strains. Separately, orthologous groups (OGs) in the 41 bacterial strains were clustered using OrthoMCL, yielding 12,354 OGs. Statistically significant associations between the CFU abundances in *D. melanogaster* and the presence-absence patterns of the OGs were identified using the R package MAGNAMWAR (Sexton *et al.* 2018). Data were processed using a linear mixed model with log-transformed CFU abundances as the response variable, and experiment and bacterial treatment as independent random effects. Statistically significant outputs were Bonferroni-corrected.

### KEGG pathway analysis

We performed a KEGG pathway analysis on the MGWA results to identify bacterial genetic pathways that were enriched among the significant MGWA results. We assigned KEGG pathway numbers to all OGs in the dataset using ‘BLASTKOALA’ and compared the number of OGs in different KEGG pathways between a reference dataset (all OGs) and a significant dataset (the top 324 significant OGs from the MGWA, based on a p-value less than 0.001) using the ‘KEGG Mapper – Search Pathway’ online tool (Kanehisa *et al.* 2017; Kanehisa and Goto 2000; Kanehisa *et al.* 2016). Significant enrichment of KEGG pathways in the top 324 significant OGs was determined by chi-square analysis, with false-discovery-rate (fdr) p-value correction in R. Pathways were only used in the analysis if they had 4 or greater counts in both the top and the reference set of OGs.

### Mutant analysis

To verify predictions of the MGWA, mutants from the arrayed library that bore lesions in LPS biosynthesis genes were reared individually with *D. melanogaster* and their load in adult flies was measured. Monoassociated *D. melanogaster* were reared as described previously (Koyle *et al.* 2016). Briefly, adult flies were reared on grape juice agar plates for 16-18 hr to allow for egg collection, eggs were collected and sterilized in two 2.5 min washes of 0.6% hypochlorite solution, rinsed three times in sterile water, and transferred to sterile yeast-glucose diet lacking preservative at a density of 30-80 eggs per vial. Selected transposon insertion mutants (see Table 1) were individually added by inoculating to the vials 50 μl of OD_600_ = 0.1 normalized bacterial culture. Three separate experiments containing all experimental treatments were performed, each in triplicate. At 5-7 days of age, pools of 5 female flies were lightly anesthetized on CO_2_ and homogenized in microcentrifuge tubes containing 125 μl MRS and 125 μl Lysing Matrix D ceramic beads (MP Biomedicals 11654034) on a GenoGrinder 2010 homogenizer for 2 min at 1250 rpm. The homogenate was dilution plated on mMRS plates and colony forming units (CFUs) were manually counted. Any flies bearing bacteria other than *Acetobacter* (determined by visual inspection of colony morphology) were removed from the experiment.

### Data and reagent availability

Strains are available upon request. File S1 contains all supplemental figures and tables together with detailed descriptions of other supplemental files. Table S1 in File S1 contains primer sequences used. Table S2 in File S1 contains data from representative conjugation experiments. Tables S3–S6 in File S1 contain pathway essentialiaty predictions for *Acetobacter fabarum DsW_054* (Table S3 in File S1), *Rhodobacter sphaeroides* (Table S4 in File S1), *Rhizobium leguminosarum* (Table S5 in File S1), and *Caulobacter crescentus* (Table S6 in File S1). Figure S1 in File S1 is a map of pJG714. File S2 is a detailed protocol for library preparation. File S3 contains the annotated insertion mutant library of *A. fabarum* DsW_054. File S4 is a script for running the MGWA analysis. File S5 contains the raw phenotype data, the means of which were originally published in (Chaston *et al*. 2014). File S6 contains the OrthoMCL gene clustering results from work initially published in (Chaston *et al*. 2014). File S7 contains the MGWA results for host colonization. File S8 describes the *Acetobacter* conjugation trials. Sequence data are available in the SRA under accession number PRJNA422683.

## Results

### Creation of an arrayed and mapped transposon insertion library in A. fabarum DsW_054

We constructed a mapped, arrayed transposon insertion library in a strain of *Acetobacter* isolated from wild *Drosophila* to enable us to study the effects of gene knockouts in an *Acetobacter* strain that was otherwise recalcitrant to genetic manipulation. We were initially unable to obtain transposon-insertion-bearing exconjugants (‘tranposants’) from matings that used as recipients two different strains of *Acetobacter* isolated from laboratory flies, *A. pomorum* DmCS_004 and *A. tropicalis* DmCS_005 (Newell and Douglas 2014). As a follow-up, we screened 17 strains of *Acetobacter* for amenability to genetic modification. Of these, *A. fabarum* DsW_054, a strain that was isolated from wild-caught *Drosophila suzukii* (Winans *et al.* 2017), yielded the greatest number of transposants with a kanamycin-marked Tn*5* transposon (Table S2 in File S1; File S8). Pairwise nucleotide alignments of the *A. fabarum* DsW_054 genome with those of two *A. fabarum* strains (KR and OG2) recently added to the NCBI WGS database, indicated >98% ANI with both, allowing us to provisionally assign this isolate to the species *fabarum* (data not shown).

*A. fabarum* DsW_054 was subjected to further optimization of a conjugation protocol to transfer a plasmid-based mini-Tn5 transposon from donor *E. coli* cells. The final protocol described in the methods was obtained after varying co-incubation ratios and times with an *E. coli* donor bearing pJG714, and selecting parameters that maximized transposant recovery. Proof-of-concept arbitrary PCR mapping confirmed that in eight randomly selected colonies, each mutant contained a single, unique transposon insertion site (data not shown).

**Figure 1:**
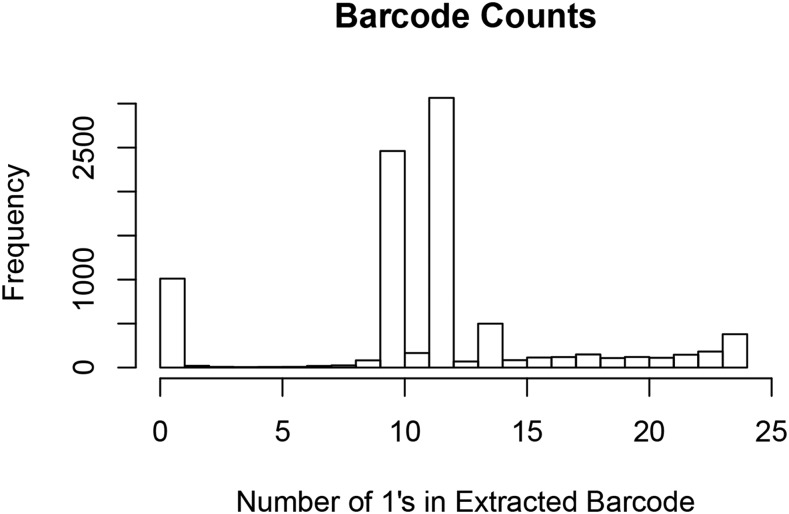
Histogram of sequencing pools in which an insertion sequence was present in the Illumina sequencing run. The combinatorial mapping barcodes assigned each mutant to 10, 12, or 14 sequencing pools, evident as the major peaks in the histogram. Any sequence that was present in anything other than 10, 12 or 14 of the 24 possible sequencing pools could not be mapped to the library and was discarded from further analysis.

We created a mapped and arrayed transposon insertion library in *A. fabarum* DsW_054, following the approach of Goodman, *et al.* (2011). 8,550 mutants were created in groups no larger than 480 mutants at a time, individually transferred to 96-well plates, and the transposon insertion sites in each arrayed mutant were defined in a single Illumina sequencing run by a combinatorial mapping approach. The insertion sites for 6,418 mutants were precisely mapped, with unmapped mutants resulting from insufficient read coverage across all sequencing pools or to duplicate insertion sites in the library (possibly from sister clones). For example, a subset of transposon insertion sites were present in more than 14 sequencing pools, most likely representing sister clones with identical insertion sites that were present in separate wells within the library; whereas the insertion sites present in fewer than 10 sequencing pools were mostly likely not abundant enough to detect in some pools (Figure 1). The relatively large number of insertion sites that were present in just 1 or 2 pools likely result from sequencing errors. To confirm the validity of the mappings, the insertion site was validated manually by arbitrary PCR and Sanger sequencing in 41 of 41 mutants selected from the library, suggesting a high level of accuracy in the mapping of the mutants to their 96-well plate arrayed location. This high validation rate suggests that most of the mutants in the library are mapped to the appropriate location.

### Prediction of essential genes in A. fabarum DsW_054

**Figure 2:**
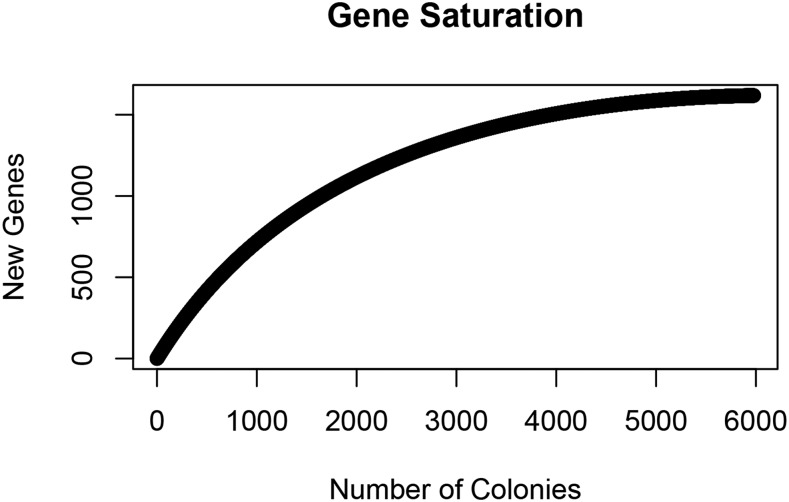
A rarefaction curve showing the number of new genes with insertions as new colonies were added to the library. The plateau suggests that the addition of mutant colonies would not add a significant number of novel genes to the mutant library.

We determined near saturation of the insertion library through analysis of hits within predicted genes. 5,559 mutants mapped within an open reading frame (ORF) called by RAST, representing insertions in 63% of the 2,579 annotated genes in the *A. fabarum* DsW_054 genome (File S3). To assess the degree of gene saturation represented by the 5,559 ORF-mapped transposon insertion mutants, we performed a rarefaction analysis (Figure 2). The plateau of the curve suggested that the mutant library was well-represented by non-essential *A. fabarum* DsW_054 genes and that mapping insertion sites in more mutants was unlikely to substantially increase the gene coverage in the collection.

Based on the gene-level saturation of the mutant library we predicted that the 37% of *A. fabarum* DsW_054 genes that did not bear any transposon insertions are essential- or are located near (*e.g.*, polar onto) essential-genes for growth in mMRS. A chi-square analysis of the KEGG pathways that were under- or over-represented in the genes bearing at least 1 transposon insertion site in our library suggested that genes encoding ribosomal components or involved in aminoacyl-tRNA biosynthesis, cell cycle, and protein export were essential for growth in mMRS; whereas the ABC transporters, starch and sucrose metabolism and two-component system pathways were enriched for non-essential genes (Table 2). Together, this analysis points to key *A. fabarum* DsW_054 genes for growth and survival in laboratory culture, the first such analysis of which we are aware for any *Acetobacteraceae* strain.

**Table 2:** Prediction of essential pathways in *Acetobacter fabarum* DsW_054

**KO**	**Pathway**	**No insertions**	**All genes**	**p-value**	**fdr corrected p-value**	**Predicted essentiality**
ko02010	ABC transporters	11	236	0.0005	0.01	nonessential
ko03010	Ribosome	46	52	0.0005	0.01	essential
ko00970	Aminoacyl-tRNA biosynthesis	22	24	0.0005	0.01	essential
ko03060	Protein export	13	19	0.0005	0.01	essential
ko04112	Cell cycle - Caulobacter	12	14	0.0005	0.01	essential
ko00500	Starch and sucrose metabolism	1	75	0.0015	0.03	nonessential
ko02020	Two-component system	15	170	0.0025	0.04	nonessential
ko00195	Photosynthesis	7	7	0.0035	0.05	essential
ko01120	Microbial metabolism in diverse environments	47	376	0.0040	0.05	nonessential
ko00330	Arginine and proline metabolism	1	53	0.0055	0.06	nonessential
ko00010	Glycolysis / Gluconeogenesis	3	66	0.0085	0.08	nonessential
ko00240	Pyrimidine metabolism	28	78	0.0110	0.09	essential
ko02040	Flagellar assembly	1	42	0.0110	0.09	nonessential
ko00780	Biotin metabolism	11	22	0.0115	0.09	essential
ko01502	Vancomycin resistance	5	5	0.0180	0.13	essential
ko01110	Biosynthesis of secondary metabolites	107	419	0.0200	0.13	essential
ko00052	Galactose metabolism	1	41	0.0210	0.130	nonessential
ko00620	Pyruvate metabolism	4	69	0.0235	0.14	nonessential
ko00550	Peptidoglycan biosynthesis	11	25	0.0360	0.20	essential
ko01230	Biosynthesis of amino acids	49	176	0.0380	0.20	essential
ko00250	Alanine, aspartate and glutamate metabolism	13	34	0.0425	0.20	essential
ko00561	Glycerolipid metabolism	1	31	0.0425	0.20	nonessential
ko00740	Riboflavin metabolism	6	10	0.0435	0.20	essential
ko02024	Quorum sensing	12	112	0.0480	0.21	nonessential
ko00400	Phenylalanine, tyrosine and tryptophan biosynthesis	13	33	0.0500	0.21	essential

Genes with no insertions were grouped into functional pathways using KEGG pathway mapper. The ‘no insertions’ column shows the number of genes that have no insertions within our library for each pathway. This was compared to the number of genes within that pathway that are present in *A. fabarum* (‘All genes’ column) using a chi-square test and the associated p-value and fdr corrected p-value are listed in addition to the predicted essentiality status for each pathway.

### Prediction of bacterial pathways that influence bacterial load in D. melanogaster

To predict bacterial genes that are necessary to associate with *D. melanogaster* we performed a MGWA analysis (File S4). The analysis was performed using previously published data from a survey of CFU abundances in *D. melanogaster* that were individually associated with each of 41 genome-sequenced bacterial strains (Chaston *et al.* 2014). An MGWA that associated bacterial CFU abundance data (File S5) with OG presence-absence patterns in the 41 strains (File S6) predicted 324 bacterial genes that influence host association using a p-value cutoff of 0.001 (File S7). Because our previous MGWA analysis had successfully identified genes by looking for enriched functions among top hits, we performed a KEGG enrichment analysis to identify pathways that were enriched in the top 324 hits from the MGWA. After correction for multiple tests, genes from just one KEGG pathway, lipopolysaccharide biosynthesis, were significantly enriched in the top MGWA hits (Table 3). Therefore, MGWA predicted a key role for bacterial LPS biosynthesis in its ability to associate with *D. melanogaster*.

**Table 3:** A KEGG pathway analysis of genes predicted to affect host colonization revealed that the LPS synthesis pathway is significant in determining bacterial abundance in the fly gut

**KO**	**Pathway**	**Top Hits**	**All**	**p-value**	**fdr**
ko00540	Lipopolysaccharide biosynthesis	9	24	0.0005	0.05
ko00270	Cysteine and methionine metabolism	7	44	0.0350	0.89
ko00480	Glutathione metabolism	4	16	0.0355	0.89
ko01501	beta-Lactam resistance	4	21	0.0600	0.89
ko03020	RNA polymerase	2	5	0.0645	0.89
ko00240	Pyrimidine metabolism	8	61	0.0725	0.89
ko00450	Selenocompound metabolism	3	14	0.0780	0.89

The top hits from the MGWA were grouped into functional pathways and compared by chi-square test to the number of all genes in that pathway from the study. The returned p-value and fdr corrected p-value are listed.

### Validation of MGWA predictions by mutant analysis

**Figure 3:**
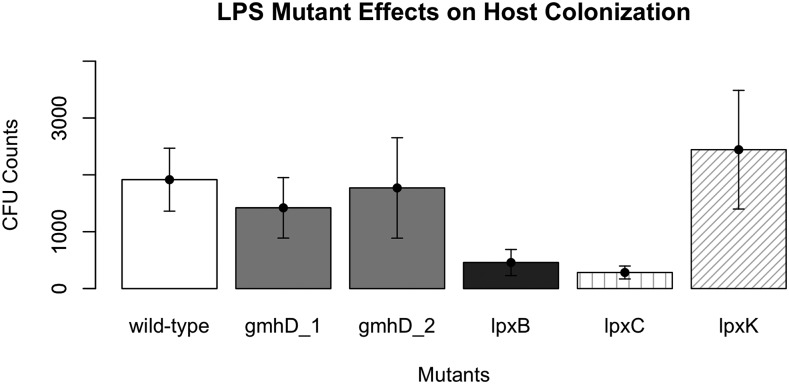
Mutant analysis of MGWA predictions. Five transposon insertion mutants for LPS biosynthesis genes were individually reared in triplicate in each of three separate experiments with *D. melanogaster*, and bacterial load was determined in whole fly homogenates when the adult flies were 5-7 days old. Significant differences between treatments were determined by a linear mixed effects model with experimental start and end date included as random effects. Insertion mutants in *lpxB* and *lpxC* were significantly less abundant in the flies than the wild-type control, but *gmhD* and *lpxK* mutations did not have a significant effect. Means and s.e.m. were derived from all replicate data points (N = 8-9 per treatment).

**Figure 4:**
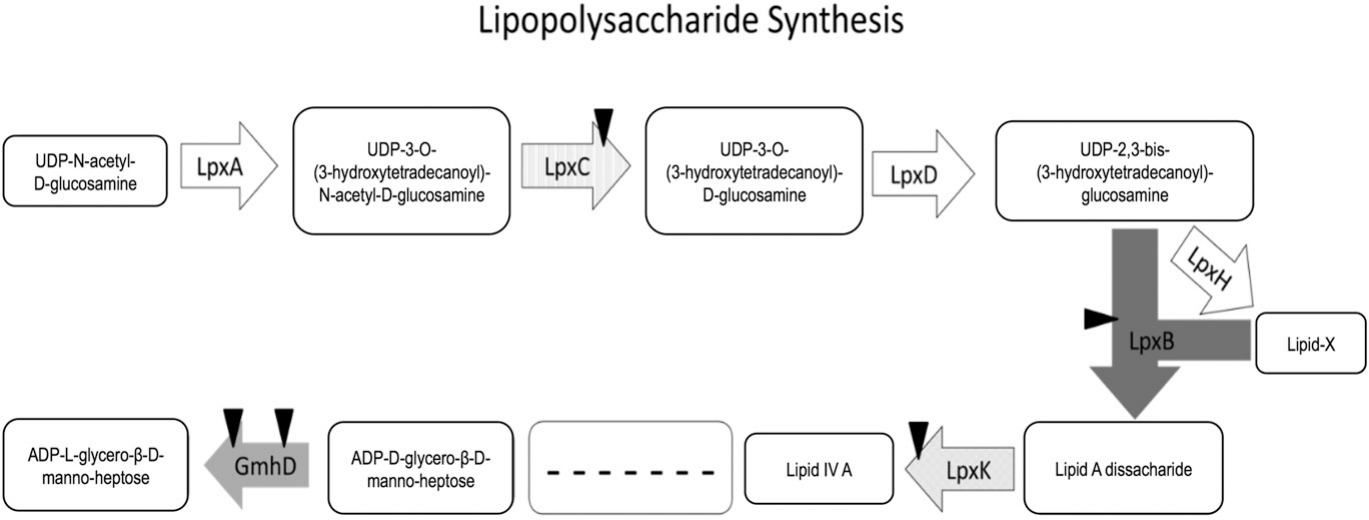
LPS biosynthesis pathway. An analysis of the LPS pathway shows that genes biosynthetically upstream, but not downstream, of Lipid A influence bacterial association with *D. melanogaster*.

Based on the MGWA predictions, we hypothesized that *A. fabarum* DsW_054 bearing lesions in LPS biosynthesis genes would have a reduced ability to associate with *D. melanogaster*. To test this hypothesis, we searched the *A. fabarum* DsW_054 transposon insertion library for all mutants corresponding to one of the KEGG LPS biosynthesis pathway genes. Five mutants including disruptions in four genes were selected for analysis, and were individually reared with sterile *D. melanogaster* eggs. CFU load was measured in 5-7 days old adults, revealing that, of the tested genes, *lpxB* and *lpxC* mutants that preceded synthesis of Lipid A disaccharide were significantly impaired in their ability to associate with the host relative to wild type *A. fabarum* DsW_054 (Figure 3). Disruption of two genes downstream of Lipid A disaccharide biosynthesis, *gmhD* and *lpxK*, including 2 distinct *gmhD* lesions, did not significantly alter the ability of *A. fabarum* DsW_054 to associate with its animal host (Figure 4). There were no differences in growth of the mutants in mMRS broth, suggesting the host association effect did not result from ‘sick’ cells (data not shown). Taken together, these results suggest an important role for *A. fabarum* DsW_054 Lipid A in *D. melanogaster* association.

## Discussion

In this study we report on an MGWA analysis to predict bacterial genes that influence the association with the fruit fly *D. melanogaster*, the creation of a mapped and arrayed transposon insertion library to identity gene-specific insertions in *A. fabarum* DsW_054, and the use of the mutant library to test the MGWA-predictions. The library includes 6,418 mutants that were mapped to 1,625 genes and 859 intergenic insertions. A 100% validation rate suggested a high accuracy rate of the mapping. Also, near-saturating coverage of non-essential genes allowed us to make inferences about which genes are essential for *A. fabarum* DsW_054 growth on mMRS medium. Finally, the host-association tests confirmed the prediction that bacterial LPS biosynthesis genes influence bacterial load in the fruit fly, and identified Lipid A biosynthesis genes as key players for these effects. Follow-up experiments that utilize the rich resources available for interrogating host-microbe interactions in *Drosophila* are necessary to characterize the molecular basis for these genetic interactions. The mutant library we report in this manuscript adds to those resources.

In this study we identified two Lipid A biosynthesis genes as important for maintaining abundant *A. fabarum* CFU loads in *D. melanogaster*, suggesting Lipid A is important for *Acetobacter* to normally associated with *D. melanogaster*. However, we note several cautions to this interpretation. First, Lipid A is generally considered to be essential for viability of most gram-negative bacteria, and viable Lipid A mutants are rare in other *Proteobacteria* (*e.g.*, *E. coli*). We have not performed any biochemical characterization of our LPS mutants and have no evidence to confirm their role on Lipid A levels in *Acetobacter*. Thus, it may be possible that these genes exert their influence independent of effects on Lipid A (*e.g.*, polarity). Alternatively, some viable LPS pathway mutants have been isolated in bacteria, such as *E. coli* (Cabeen *et al.* 2010; Emptage *et al.* 2014; Fuhrer *et al.* 2006; Karsten *et al.* 2009; Kuo *et al.* 2016; Langklotz *et al.* 2011; Powell *et al.* 2016; Vorachek-Warren *et al.* 2002; Vuorio and Vaara 1992, 1995; Wang *et al.* 2014), including complete deletion of *gmhD* (*rfaD*) (Wang *et al.* 2014), temperature-sensitive *lpxA* and *lpxD* mutations (Vuorio and Vaara 1992, 1995), and an *lpxC* gene truncation (Fuhrer *et al.* 2006; Langklotz *et al.* 2011). Mutations of other genes involved in Lipid A modification include *lpxM*, *lpxP*, *lpxL* and many genes involved in the formation of O antigen (Cabeen *et al.* 2010; Emiola *et al.* 2014; Karsten *et al.* 2009; Powell *et al.* 2016; Vorachek-Warren *et al.* 2002).

Despite these caveats associated with a possible LPS effect, there are strong established relationships between bacterial LPS and animal colonization. Consistent with our current findings, the structure of Lipid A in *Francisella tularensis* is important in bacterial resistance to fruit fly anti-microbial peptides, and a Lipid A core mutant persisted in lower CFU loads during infection than a wild-type control strain (Vonkavaara *et al.* 2013). Although the Toll and IMD immune pathways in *Drosophila* do not respond to LPS, *Drosophila* can sense LPS through neuron stimulation, allowing for pathogen protection and infection avoidance. It is hypothesized that this occurs through taste as flies eat, allowing them to avoid unwanted bacteria (Soldano *et al.* 2016), and LPS causes an increase in serotonin production in insects that increases phagocytosis by hemocytes and improves the insect’s ability to fight infection (Qi *et al.* 2016). LPS is also important for bacterial persistence in other animals. For example, a TnSeq experiment of *Snodgrassella alvi* colonization in honey bees also revealed that LPS is an important factor in colonization (Powell *et al.* 2016). In non-insect hosts, *E. coli* LPS mutants are hyper-susceptible to host immunity in *C. elegans* ((Kuo *et al.* 2016)), and LPS is important for *E. coli* colonization and persistence in sheep (Cornick *et al.* 2017). *Vibrio cholerae* LPS mutants had a 30 fold reduction in colonization of the mouse gut (Nesper *et al.* 2002). Inactivation of PA0011 (involved in Lipid A biosynthesis) in *Pseudomonas aeruginosa* caused decreased virulence and increased susceptibility to antibiotics (Wang *et al.* 2016a). Thus, our findings are consistent with a broad base of literature that has established LPS biosynthesis is important for host association across the animal kingdom, even though the mechanisms may vary. The postulated explanation in other animals – that LPS is recognized by the host innate immune system to recognize and defend against potential pathogens prior to infection (Charroux *et al.* 2009; Kurata 2014) – may not apply in fruit flies since LPS does not appear to stimulate immune activity in *Drosophila* (Kaneko *et al.* 2004; Leulier *et al.* 2003). We propose at least three possible explanations for how mutations in LPS biosynthesis could influence bacterial load in the flies. First, the mutations may reduce bacterial growth when the fly is present, even though there was no defect in bacterial growth in 2xYPG. Second, the mutations may lead to weaker cell membranes that are more sensitive to digestion (Vorachek-Warren *et al.* 2002; Vuorio and Vaara 1992). Third, the bacteria may influence fly feeding or other behavior preferences (Kim *et al.* 2017; Fischer *et al.* 2017) that consequently alter bacterial load. Future experiments are necessary to definitively test these ideas.

An analysis of essential genes in an *Acetobacteraceae* strain has not been determined previously. A method that samples more deeply than our study is necessary for a comprehensive reporting of essential *A. fabarum* DsW_054 genes (*e.g.*, by TnSeq), but the saturation of non-essential genes as estimated by a rarefaction curve (Figure 2) suggests some preliminary insights can be gleaned from our work, including that ribosome, aminoacyl tRNA biosynthesis, protein export and cell cycle functions are likely essential for *A. fabarum* DsW_054 growth in mMRS. As a secondary confirmation for this prediction, we detected congruence between these predictions and TnSeq analyses of the closest *A. fabarum* DsW_054 relatives for which a study had been performed: *Alphaproteobacteria Caulobacter crescentus* (Christen *et al.* 2011), *Rhizobium leguminosarum* (Perry *et al.* 2016) and *Rhodobacter sphaeroides* (Burger *et al.* 2017). Chi-square tests on the KEGG pathways enriched in predicted essential genes from these three studies confirmed essential roles for ribosome and Aminoacyl-tRNA biosynthesis in all three organisms, along with nonsignificant but trending enrichment for cell cycle and metabolic pathways (see Tables S3–S6 in File S1). One limitation of our analysis *A. fabarum* DsW_054 is that any gene with at least one insertion is binned as non-essential; this categorization does not take into account genes that are only partially disabled by transposon insertion (*e.g.*, into the 3′ end of a gene) that could be assigned as essential. Regardless, consistency between the essential genes detected in our study and in other conphyletic taxa lend support to these cautious interpretations.

The arrayed and mapped *A. fabarum* DsW_054 transposon insertion library we created in this study will be a resource for use in any field that studies *Acetobacter* genetics. *Acetobacter* species are studied in a diversity of research fields, including as members of insect microbiomes and in commercial production facilities (*e.g.*, fermentation, cellulose production). We focused on *Acetobacter* as representative members of the *Drosophila* gut microbiota, and previous studies have identified species-specific influence of *Acetobacter* strains on numerous traits, including triacylglyeride and glucose content, metabolic rate, development time, fecundity, dietary choices, and egg laying preferences (Fischer *et al.* 2017; Chaston *et al.* 2014; Ridley *et al.* 2012; Morimoto *et al.* 2017; Leitão-Gonçalves *et al.* 2017). *Acetobacter* are also commonly associated with numerous other insect species, including honey bees (*Apis mellifera*), *Anopheles* and *Aedes* mosquitos, leafhoppers (*Scaphoideus titanus*), and mealybugs (*Saccharicoccus sacchari*) (Crotti *et al.* 2010). *Acetobacter* species are also important in commercial production of acetic acid or fermentation products, such as Kefir, a fermented beverage (Cleenwerck *et al.* 2008; Garofalo *et al.* 2015; Gulitz *et al.* 2011; Moens *et al.* 2014; Viana *et al.* 2017; Wang *et al.* 2016b). *Acetobacter* species have also been used in other applications in biotechnology, including microbial fuel cell technology to produce both electricity and acetic acid (Tanino *et al.* 2013), in the production of bacterial cellulose scaffolds to grow cartilage and skin tissue (Yin *et al*. 2015, Keskin *et al*. 2017) and is a hopeful candidate for dermal medical applications (Taokaew *et al.* 2015). As such, this mutant library has the potential to serve as a resource for numerous areas of research into the genetic basis for any of these *Acetobacter* applications, and we welcome requests for specific strains of interest (see File S3 for full list).

## Supplementary Material

Supplemental Material is available online at www.g3journal.org/lookup/suppl/doi:10.1534/g3.117.300530/-/DC1.
