# Supplementary material for: A Metagenome-Wide Association Study and Arrayed Mutant Library Confirm Acetobacter Lipopolysaccharide Genes Are Necessary for Association with Drosophila melanogaster
Source: G3 (Bethesda). 2018 Feb 27;8(4):1119–27. doi: 10.1534/g3.117.300530 (PMC5873903; doi:10.1534/g3.117.300530)
Supplement: Supplementary file 1 [file 1119FileS1.docx]

**Supplementary file 1**

***In this document***

**Table S1**: **Primer sequences.**

**Table S2: Data from representative conjugation experiments**. The number of exconjugates per conjugation was calculated from CFU on selective plates. Each conjugation began with approximately 5 x 10^6^ recipient cells. SR = spontaneous resistance was common in the negative control in which recipient was incubated without a donor and plated on selective media, effectively nullifying the conjugation results. All strains from Winans et al. 2017 unless indicated.

**Table S3:** **Pathway essentiality predictions of *Acetobacter fabarum DsW_054***. ‘KO’ column gives the KEGG number assignment and the associated functional pathway is listed in the ‘pathway’ column. The ‘All’ column lists the number of genes within *A. fabarum* that are part of the associated pathway, regardless of essentiality status while the ‘Essential genes’ lists the number of genes within *A. fabarum* that had no insertion mutants in our library within the associated pathway. The p-values from the chi-square test and the FDR correction are listed in the next columns. The predicted essentiality status of the pathway is listed in the final column.

**Table S4: Pathway essentiality predictions of *Rhodobacter sphaeroides***. ‘KO’ column gives the KEGG number assignment and the associated functional pathway is listed in the ‘pathway’ column. The ‘All’ column lists the number of genes within *R. sphaeroides* that are part of the associated pathway, regardless of essentiality status while the ‘Essential genes’ lists the number of genes within *R. sphaeroides* that are predicted to be essential within the associated pathway. The p-values from the chi-square test and the FDR correction are listed in the next columns. The predicted essentiality status of the pathway is listed in the final column.

**Table S5**: **Pathway essentiality predictions of *Rhizobium leguminosarum*.** ‘Pathway’ column gives the KEGG number assignment and the associated functional pathway. The ‘All’ column lists the number of genes within *R. leguminosarum* that are part of the associated pathway, regardless of essentiality status while the ‘Essential genes’ lists the number of genes within *R.* *leguminosarum* that are predicted to be essential within the associated pathway. The p-values from the chi-square test and the FDR correction are listed in the next columns. The predicted essentiality status of the pathway is listed in the final column.

**Table S6: Pathway essentiality predictions of *Caulobacter crescentus***. ‘KO’ column gives the KEGG number assignment and the associated functional pathway is listed in the ‘pathway’ column. The ‘All’ column lists the number of genes within C*. crescentus* that are part of the associated pathway, regardless of essentiality status while the ‘Essential genes’ lists the number of genes within *C.* *crescentus* that are predicted to be essential within the associated pathway. The p-values from the chi-square test and the FDR correction are listed in the next columns. The predicted essentiality status of the pathway is listed in the final column.

**Figure S1: Transposon delivery vector pJG714 and amplification strategy for deep sequencing of transposon insertion sites.** (A) Map of pJG714. Regions encoding the Tn5 transposase (tnp), kanamycin resistance gene (kan/neo), *Salmonella* trp promoter (Ptrp), conjugal transfer origin (oriT), pir-dependent replication origin (oriV), and transposon ends (TE) are indicated. (B) Diagram outlining the Illumina library construction method, including enzymatic fragmentation, tailing with poly-C, and PCR-mediated introduction of Illumina adapters (grey bars) and sample-specific barcodes (bar).

***Not in this document***

**File S2: Library preparation protocol**: DNA from combinatorial mapping pools was prepared for sequencing using the following protocol.

**File S3. Annotations of the A. fabarum arrayed mutant library.** All genes and their associated insertion mutants are listed with exact insertion sites and the assigned KO #.

**File S4: Script to perform MGWA.** Data from these files in a different format were originally published in PMID 25271286.

**File S5: MGWA phenotypes file.** Raw counts of colony forming units per fly. Mean values were previously published in PMID 25271286.

**File S6: MGWA Genotypes file.** Groups file produced by clustering genomes using OrthoMCL. Data are from PMID 25271286.

**File S7:** **Results of the MGWA for host colonization abundance.** Data were collected from homogenized flies that had been mono-associated with 41 different bacterial strains and species. The differences were compared based on gene presence/absence within the associated microbes to produce genes predicted to affect abundance.

**File S8: Description of *Acetobacter* Conjugation Trials**

**Table S1**: **Primer sequences**

| **Primer** | **Sequence** |
| --- | --- |
| 1TN | CTGACCCGGTCGAC |
| 1OLIGOG | CAGACGTGTGCTCTTCCGATCggggggggggg |
| 2BAR01 | CAAGCAGAAGACGGCATACGAGAT*ATCACG*GTGACTGGAGTTCAGACGTGTGCTCTTCCGATC |
| 2BAR02: | CAAGCAGAAGACGGCATACGAGAT*CGATGT*GTGACTGGAGTTCAGACGTGTGCTCTTCCGATC |
| 2BAR03: | CAAGCAGAAGACGGCATACGAGAT*TTAGGC*GTGACTGGAGTTCAGACGTGTGCTCTTCCGATC |
| 2BAR04: | CAAGCAGAAGACGGCATACGAGAT*GCCAAT*GTGACTGGAGTTCAGACGTGTGCTCTTCCGATC |
| 2BAR05: | CAAGCAGAAGACGGCATACGAGAT*ACAGTG*GTGACTGGAGTTCAGACGTGTGCTCTTCCGATC |
| 2BAR06: | CAAGCAGAAGACGGCATACGAGAT*CAGATC*GTGACTGGAGTTCAGACGTGTGCTCTTCCGATC |
| 2BAR07: | CAAGCAGAAGACGGCATACGAGAT*TGACCA*GTGACTGGAGTTCAGACGTGTGCTCTTCCGATC |
| 2BAR08: | CAAGCAGAAGACGGCATACGAGAT*GATCAG*GTGACTGGAGTTCAGACGTGTGCTCTTCCGATC |
| 2BAR09: | CAAGCAGAAGACGGCATACGAGAT*ACTTGA*GTGACTGGAGTTCAGACGTGTGCTCTTCCGATC |
| 2BAR10: | CAAGCAGAAGACGGCATACGAGAT*CTTGTA*GTGACTGGAGTTCAGACGTGTGCTCTTCCGATC |
| 2BAR11: | CAAGCAGAAGACGGCATACGAGAT*TAGCTT*GTGACTGGAGTTCAGACGTGTGCTCTTCCGATC |
| 2BAR12: | CAAGCAGAAGACGGCATACGAGAT*GGCTAC*GTGACTGGAGTTCAGACGTGTGCTCTTCCGATC |
| 2BAR13: | CAAGCAGAAGACGGCATACGAGAT*AGTCAA*GTGACTGGAGTTCAGACGTGTGCTCTTCCGATC |
| 2BAR14: | CAAGCAGAAGACGGCATACGAGAT*CGTACG*GTGACTGGAGTTCAGACGTGTGCTCTTCCGATC |
| 2BAR15: | CAAGCAGAAGACGGCATACGAGAT*TAATCG*GTGACTGGAGTTCAGACGTGTGCTCTTCCGATC |
| 2BAR16: | CAAGCAGAAGACGGCATACGAGAT*GTAGAG*GTGACTGGAGTTCAGACGTGTGCTCTTCCGATC |
| 2BAR17: | CAAGCAGAAGACGGCATACGAGAT*AGTTCC*GTGACTGGAGTTCAGACGTGTGCTCTTCCGATC |
| 2BAR18: | CAAGCAGAAGACGGCATACGAGAT*CAACTA*GTGACTGGAGTTCAGACGTGTGCTCTTCCGATC |
| 2BAR19: | CAAGCAGAAGACGGCATACGAGAT*TACAGC*GTGACTGGAGTTCAGACGTGTGCTCTTCCGATC |
| 2BAR20: | CAAGCAGAAGACGGCATACGAGAT*GAGTGG*GTGACTGGAGTTCAGACGTGTGCTCTTCCGATC |
| 2BAR21: | CAAGCAGAAGACGGCATACGAGAT*ATGTCA*GTGACTGGAGTTCAGACGTGTGCTCTTCCGATC |
| 2BAR22: | CAAGCAGAAGACGGCATACGAGAT*CACGAT*GTGACTGGAGTTCAGACGTGTGCTCTTCCGATC |
| 2BAR23: | CAAGCAGAAGACGGCATACGAGAT*TCATTC*GTGACTGGAGTTCAGACGTGTGCTCTTCCGATC |
| 2BAR24: | CAAGCAGAAGACGGCATACGAGAT*GGTAGC*GTGACTGGAGTTCAGACGTGTGCTCTTCCGATC |
| 2TNA | AATGATACGGCGACCACCGAGATCTACACTCTTTCCCTACACGACGCTCTTCCGATCTnTCGAGATGTGTATAAGAGACAG |
| 2TNB | AATGATACGGCGACCACCGAGATCTACACTCTTTCCCTACACGACGCTCTTCCGATCTnnTCGAGATGTGTATAAGAGACAG |
| 2TNC | AATGATACGGCGACCACCGAGATCTACACTCTTTCCCTACACGACGCTCTTCCGATCTnnnTCGAGATGTGTATAAGAGACAG |
| 2TND | AATGATACGGCGACCACCGAGATCTACACTCTTTCCCTACACGACGCTCTTCCGATCTnnnnTCGAGATGTGTATAAGAGACAG |
| Arb1 | GGCCACGCGTCGACTAGTACNNNNNNNNNNGATAT |
| Arb6 | GGCCACGCGTCGACTAGTACNNNNNNNNNNACGCC |
| Arb2 | GGCCACGCGTCGACTAGTAC |
| 133 | GTTTACTTTGCAGGGCTTCCCAAC |
| 134 | AGCTGGCAATTCCGGTTCGCTTG |

**Table S2: Data from representative conjugation experiments**

|  | **Exconjugates per conjugation** | |
| --- | --- | --- |
| **Recipient Strain** | **pCM62 (tet)** | **pRL27 (Tn5 Kan)** |
| *Acetobacter tropicalis* SKU1100^a^ | 2.50E+03 | SR |
| *Acetobacter pasteurianus* SKU1108^a^ | 7.50E+03 | 0.00E+00 |
| *Acetobacter pasteurianus* NBRC 106471^a^ | 4.90E+05 | 1.00E+03 |
| *Acetobacter pasteurianus* 3p3^b^ | 5.60E+05 | 2.25E+04 |
| *Acetobacter tropicalis* DmW_042 | 5.05E+05 | 1.70E+05 |
| *Acetobacter sp.* DmW_043 | 7.80E+05 | SR |
| *Acetobacter cibinongensis* DmW_047 | 6.00E+04 | SR |
| *Acetobacter tropicalis* DmL_050 | 1.20E+06 | 3.45E+04 |
| *Acetobacter persici* DmL_053 | 9.75E+05 | SR |
| *Acetobacter fabarum* DsW_054 | 1.15E+06 | 7.00E+05 |
| *Acetobacter malorum* DsW_057 | 0.00E+00 | 0.00E+00 |
| *Acetobacter sp.* DsW_059 | 1.15E+06 | 2.00E+05 |

a) Courtesy of K. Matsushita, Yamaguchi University, Japan.

b) Courtesy of F. Barja, University of Geneva, Switzerland.

**Table S3:** **Pathway essentiality predictions of *Acetobacter fabarum DsW_054***

| KO | Pathway | Essential Genes | All | p-value | fdr | Predicted essentiality |
| --- | --- | --- | --- | --- | --- | --- |
| ko02010 | ABC transporters | 11 | 236 | 0.0005 | 0.01 | nonessential |
| ko03010 | Ribosome | 46 | 52 | 0.0005 | 0.01 | essential |
| ko00970 | Aminoacyl-tRNA biosynthesis | 22 | 24 | 0.0005 | 0.01 | essential |
| ko03060 | Protein export | 13 | 19 | 0.0005 | 0.01 | essential |
| ko04112 | Cell cycle - Caulobacter | 12 | 14 | 0.0005 | 0.01 | essential |
| ko02020 | Two-component system | 15 | 170 | 0.0015 | 0.03 | nonessential |
| ko00500 | Starch and sucrose metabolism | 1 | 75 | 0.0030 | 0.05 | nonessential |
| ko00195 | Photosynthesis | 7 | 7 | 0.0035 | 0.05 | essential |
| ko00010 | Glycolysis / Gluconeogenesis | 3 | 66 | 0.0040 | 0.05 | nonessential |
| ko00330 | Arginine and proline metabolism | 1 | 53 | 0.0060 | 0.06 | nonessential |
| ko01120 | Microbial metabolism in diverse environments | 47 | 376 | 0.0075 | 0.07 | nonessential |
| ko02040 | Flagellar assembly | 1 | 42 | 0.0090 | 0.08 | nonessential |
| ko00240 | Pyrimidine metabolism | 28 | 78 | 0.0110 | 0.08 | essential |
| ko00620 | Pyruvate metabolism | 4 | 69 | 0.0125 | 0.08 | nonessential |
| ko00780 | Biotin metabolism | 11 | 22 | 0.0120 | 0.08 | essential |
| ko01502 | Vancomycin resistance | 5 | 5 | 0.0120 | 0.08 | essential |
| ko00052 | Galactose metabolism | 1 | 41 | 0.0135 | 0.08 | nonessential |
| ko01110 | Biosynthesis of secondary metabolites | 107 | 419 | 0.0210 | 0.12 | essential |
| ko00550 | Peptidoglycan biosynthesis | 11 | 25 | 0.0260 | 0.14 | essential |
| ko01230 | Biosynthesis of amino acids | 49 | 176 | 0.0325 | 0.17 | essential |
| ko00740 | Riboflavin metabolism | 6 | 10 | 0.0365 | 0.18 | essential |
| ko00400 | Phenylalanine, tyrosine and tryptophan biosynthesis | 13 | 33 | 0.0430 | 0.21 | essential |
| ko02024 | Quorum sensing | 12 | 112 | 0.0520 | 0.21 | nonessential |
| ko00230 | Purine metabolism | 31 | 105 | 0.0520 | 0.21 | essential |
| ko00250 | Alanine, aspartate and glutamate metabolism | 13 | 34 | 0.0510 | 0.21 | essential |
| ko00561 | Glycerolipid metabolism | 1 | 31 | 0.0525 | 0.21 | nonessential |
| ko00360 | Phenylalanine metabolism | 2 | 40 | 0.0555 | 0.22 | nonessential |
| ko00300 | Lysine biosynthesis | 10 | 25 | 0.0630 | 0.24 | essential |
| ko03020 | RNA polymerase | 4 | 6 | 0.0680 | 0.25 | essential |
| ko00640 | Propanoate metabolism | 3 | 44 | 0.0750 | 0.26 | nonessential |
| ko05111 | Biofilm formation - Vibrio cholerae | 1 | 27 | 0.0760 | 0.26 | nonessential |
| ko00473 | D-Alanine metabolism | 3 | 4 | 0.0935 | 0.30 | essential |
| ko00471 | D-Glutamine and D-glutamate metabolism | 3 | 4 | 0.0920 | 0.30 | essential |
| ko02026 | Biofilm formation - Escherichia coli | 3 | 40 | 0.1000 | 0.31 | nonessential |
| ko00350 | Tyrosine metabolism | 2 | 33 | 0.1039 | 0.31 | nonessential |
| ko00190 | Oxidative phosphorylation | 20 | 68 | 0.1119 | 0.33 | essential |
| ko00061 | Fatty acid biosynthesis | 10 | 27 | 0.1194 | 0.34 | essential |
| ko00051 | Fructose and mannose metabolism | 5 | 55 | 0.1229 | 0.34 | nonessential |
| ko00380 | Tryptophan metabolism | 1 | 22 | 0.1379 | 0.37 | nonessential |
| ko00910 | Nitrogen metabolism | 2 | 29 | 0.1554 | 0.41 | nonessential |
| ko00410 | beta-Alanine metabolism | 1 | 23 | 0.1654 | 0.41 | nonessential |
| ko02025 | Biofilm formation - Pseudomonas aeruginosa | 1 | 21 | 0.1654 | 0.41 | nonessential |
| ko00053 | Ascorbate and aldarate metabolism | 1 | 21 | 0.1599 | 0.41 | nonessential |
| ko03030 | DNA replication | 9 | 27 | 0.1749 | 0.41 | essential |
| ko00900 | Terpenoid backbone biosynthesis | 7 | 19 | 0.1729 | 0.41 | essential |
| ko03018 | RNA degradation | 7 | 19 | 0.1794 | 0.41 | essential |
| ko01130 | Biosynthesis of antibiotics | 72 | 303 | 0.1944 | 0.44 | essential |
| ko01200 | Carbon metabolism | 23 | 156 | 0.2154 | 0.46 | nonessential |
| ko00520 | Amino sugar and nucleotide sugar metabolism | 9 | 72 | 0.2194 | 0.46 | nonessential |
| ko00030 | Pentose phosphate pathway | 6 | 54 | 0.2199 | 0.46 | nonessential |
| ko00040 | Pentose and glucuronate interconversions | 2 | 26 | 0.2104 | 0.46 | nonessential |
| ko01523 | Antifolate resistance | 2 | 4 | 0.2364 | 0.48 | essential |
| ko00630 | Glyoxylate and dicarboxylate metabolism | 5 | 45 | 0.2559 | 0.51 | nonessential |
| ko00785 | Lipoic acid metabolism | 2 | 4 | 0.2644 | 0.52 | essential |
| ko01100 | Metabolic pathways | 225 | 1051 | 0.2944 | 0.56 | essential |
| ko03430 | Mismatch repair | 10 | 33 | 0.2919 | 0.56 | essential |
| ko00650 | Butanoate metabolism | 5 | 42 | 0.3318 | 0.62 | nonessential |
| ko00680 | Methane metabolism | 6 | 49 | 0.3618 | 0.63 | nonessential |
| ko00564 | Glycerophospholipid metabolism | 6 | 48 | 0.3638 | 0.63 | nonessential |
| ko00750 | Vitamin B6 metabolism | 5 | 14 | 0.3498 | 0.63 | essential |
| ko00670 | One carbon pool by folate | 5 | 14 | 0.3623 | 0.63 | essential |
| ko00920 | Sulfur metabolism | 6 | 46 | 0.3698 | 0.63 | nonessential |
| ko00730 | Thiamine metabolism | 2 | 21 | 0.3973 | 0.67 | nonessential |
| ko01212 | Fatty acid metabolism | 10 | 38 | 0.4253 | 0.69 | essential |
| ko00460 | Cyanoamino acid metabolism | 3 | 9 | 0.4228 | 0.69 | essential |
| ko00760 | Nicotinate and nicotinamide metabolism | 3 | 27 | 0.4728 | 0.74 | nonessential |
| ko00340 | Histidine metabolism | 7 | 25 | 0.4673 | 0.74 | essential |
| ko00627 | Aminobenzoate degradation | 1 | 14 | 0.4828 | 0.74 | nonessential |
| ko00982 | Drug metabolism - cytochrome P450 | 1 | 13 | 0.4778 | 0.74 | nonessential |
| ko00980 | Metabolism of xenobiotics by cytochrome P450 | 1 | 13 | 0.4928 | 0.75 | nonessential |
| ko01210 | 2-Oxocarboxylic acid metabolism | 5 | 37 | 0.5452 | 0.81 | nonessential |
| ko00020 | Citrate cycle | 5 | 35 | 0.5512 | 0.81 | nonessential |
| ko03070 | Bacterial secretion system | 11 | 46 | 0.5727 | 0.83 | essential |
| ko01503 | Cationic antimicrobial peptide | 3 | 22 | 0.6057 | 0.87 | nonessential |
| ko04070 | Phosphatidylinositol signaling system | 2 | 5 | 0.6302 | 0.89 | essential |
| ko00860 | Porphyrin and chlorophyll metabolism | 11 | 49 | 0.7171 | 0.89 | essential |
| ko00720 | Carbon fixation pathways in prokaryotes | 6 | 37 | 0.7041 | 0.89 | nonessential |
| ko01501 | beta-Lactam resistance | 4 | 29 | 0.6427 | 0.89 | nonessential |
| ko00983 | Drug metabolism - other enzymes | 3 | 11 | 0.7046 | 0.89 | essential |
| ko03420 | Nucleotide excision repair | 1 | 11 | 0.7071 | 0.89 | nonessential |
| ko00960 | Tropane, piperidine and pyridine alkaloid biosynthesis | 1 | 11 | 0.7031 | 0.89 | nonessential |
| ko00521 | Streptomycin biosynthesis | 1 | 11 | 0.6937 | 0.89 | nonessential |
| ko00333 | Prodigiosin biosynthesis | 3 | 10 | 0.7046 | 0.89 | essential |
| ko00600 | Sphingolipid metabolism | 1 | 9 | 0.7101 | 0.89 | nonessential |
| ko00430 | Taurine and hypotaurine metabolism | 1 | 9 | 0.6937 | 0.89 | nonessential |
| ko05418 | Fluid shear stress and atherosclerosis | 3 | 12 | 0.7356 | 0.91 | essential |
| ko04146 | Peroxisome | 2 | 15 | 0.7456 | 0.91 | nonessential |
| ko00710 | Carbon fixation in photosynthetic organisms | 5 | 21 | 0.7916 | 0.91 | essential |
| ko00130 | Ubiquinone and other terpenoid-quinone biosynthesis | 5 | 20 | 0.7766 | 0.91 | essential |
| ko04122 | Sulfur relay system | 3 | 20 | 0.7721 | 0.91 | nonessential |
| ko00310 | Lysine degradation | 3 | 19 | 0.7931 | 0.91 | nonessential |
| ko03410 | Base excision repair | 2 | 15 | 0.7631 | 0.91 | nonessential |
| ko00770 | Pantothenate and CoA biosynthesis | 7 | 30 | 0.8161 | 0.92 | essential |
| ko00480 | Glutathione metabolism | 6 | 27 | 0.8171 | 0.92 | essential |
| ko00260 | Glycine, serine and threonine metabolism | 11 | 62 | 0.8701 | 0.97 | nonessential |
| ko00270 | Cysteine and methionine metabolism | 11 | 60 | 0.8751 | 0.97 | nonessential |
| ko03440 | Homologous recombination | 7 | 34 | 1.0000 | 1.00 | essential |
| ko02030 | Bacterial chemotaxis | 6 | 29 | 1.0000 | 1.00 | essential |
| ko00220 | Arginine biosynthesis | 6 | 28 | 1.0000 | 1.00 | essential |
| ko00540 | Lipopolysaccharide biosynthesis | 5 | 27 | 1.0000 | 1.00 | nonessential |
| ko00450 | Selenocompound metabolism | 3 | 17 | 1.0000 | 1.00 | nonessential |
| ko00261 | Monobactam biosynthesis | 3 | 15 | 1.0000 | 1.00 | essential |
| ko01040 | Biosynthesis of unsaturated fatty acids | 2 | 13 | 1.0000 | 1.00 | nonessential |
| ko00562 | Inositol phosphate metabolism | 2 | 12 | 1.0000 | 1.00 | nonessential |
| ko01524 | Platinum drug resistance | 2 | 11 | 1.0000 | 1.00 | nonessential |
| ko00401 | Novobiocin biosynthesis | 2 | 10 | 1.0000 | 1.00 | essential |

**Table S4: Pathway essentiality predictions of *Rhodobacter sphaeroides***

| KO | Pathway | Essential genes | All | p-value | fdr | Predicted essentiality |
| --- | --- | --- | --- | --- | --- | --- |
| ko02010 | ABC transporters | 13 | 202 | 0.0005 | 0.03 | nonessential |
| ko02020 | Two-component system | 9 | 134 | 0.0005 | 0.03 | nonessential |
| ko03010 | Ribosome | 36 | 54 | 0.0005 | 0.03 | essential |
| ko02024 | Quorum sensing | 9 | 125 | 0.0010 | 0.03 | nonessential |
| ko02040 | Flagellar assembly | 0 | 60 | 0.0010 | 0.03 | nonessential |
| ko00970 | Aminoacyl-tRNA biosynthesis | 24 | 27 | 0.0005 | 0.03 | essential |
| ko03440 | Homologous recombination | 17 | 20 | 0.0010 | 0.03 | essential |
| ko02030 | Bacterial chemotaxis | 0 | 47 | 0.0010 | 0.03 | nonessential |
| ko03030 | DNA replication | 12 | 16 | 0.0015 | 0.04 | essential |
| ko00540 | Lipopolysaccharide biosynthesis | 10 | 11 | 0.0035 | 0.08 | essential |
| ko00190 | Oxidative phosphorylation | 31 | 72 | 0.0040 | 0.08 | essential |
| ko00630 | Glyoxylate and dicarboxylate metabolism | 3 | 57 | 0.0070 | 0.11 | nonessential |
| ko00550 | Peptidoglycan biosynthesis | 12 | 19 | 0.0060 | 0.11 | essential |
| ko00061 | Fatty acid biosynthesis | 13 | 21 | 0.0070 | 0.11 | essential |
| ko01100 | Metabolic pathways | 199 | 695 | 0.0105 | 0.13 | essential |
| ko00680 | Methane metabolism | 0 | 32 | 0.0095 | 0.13 | nonessential |
| ko03060 | Protein export | 11 | 17 | 0.0100 | 0.13 | essential |
| ko04112 | Cell cycle - Caulobacter | 11 | 19 | 0.0160 | 0.19 | essential |
| ko00240 | Pyrimidine metabolism | 21 | 50 | 0.0175 | 0.20 | essential |
| ko00500 | Starch and sucrose metabolism | 0 | 25 | 0.0180 | 0.20 | nonessential |
| ko00330 | Arginine and proline metabolism | 0 | 27 | 0.0225 | 0.23 | nonessential |
| ko00010 | Glycolysis / Gluconeogenesis | 1 | 33 | 0.0275 | 0.25 | nonessential |
| ko01212 | Fatty acid metabolism | 13 | 27 | 0.0285 | 0.25 | essential |
| ko00300 | Lysine biosynthesis | 10 | 18 | 0.0275 | 0.25 | essential |
| ko00740 | Riboflavin metabolism | 6 | 7 | 0.0260 | 0.25 | essential |
| ko00340 | Histidine metabolism | 0 | 20 | 0.0315 | 0.26 | nonessential |
| ko00280 | Valine, leucine and isoleucine degradation | 1 | 31 | 0.0375 | 0.30 | nonessential |
| ko00860 | Porphyrin and chlorophyll metabolism | 21 | 54 | 0.0420 | 0.33 | essential |
| ko03070 | Bacterial secretion system | 11 | 23 | 0.0440 | 0.33 | essential |
| ko00195 | Photosynthesis | 8 | 15 | 0.0560 | 0.41 | essential |
| ko03430 | Mismatch repair | 10 | 22 | 0.0635 | 0.44 | essential |
| ko02025 | Biofilm formation - Pseudomonas aeruginosa | 0 | 18 | 0.0660 | 0.44 | nonessential |
| ko01502 | Vancomycin resistance | 4 | 5 | 0.0645 | 0.44 | essential |
| ko00130 | Ubiquinone and other terpenoid-quinone biosynthesis | 5 | 8 | 0.0685 | 0.44 | essential |
| ko04260 | Cardiac muscle contraction | 3 | 3 | 0.0735 | 0.46 | essential |
| ko00020 | Citrate cycle | 11 | 26 | 0.0940 | 0.57 | essential |
| ko00620 | Pyruvate metabolism | 5 | 47 | 0.1014 | 0.58 | nonessential |
| ko00220 | Arginine biosynthesis | 1 | 21 | 0.0995 | 0.58 | nonessential |
| ko03020 | RNA polymerase | 3 | 4 | 0.1204 | 0.67 | essential |
| ko00900 | Terpenoid backbone biosynthesis | 8 | 18 | 0.1269 | 0.69 | essential |
| ko00920 | Sulfur metabolism | 2 | 26 | 0.1474 | 0.75 | nonessential |
| ko00052 | Galactose metabolism | 0 | 12 | 0.1449 | 0.75 | nonessential |
| ko00350 | Tyrosine metabolism | 0 | 11 | 0.1429 | 0.75 | nonessential |
| ko00051 | Fructose and mannose metabolism | 2 | 27 | 0.1589 | 0.79 | nonessential |
| ko00785 | Lipoic acid metabolism | 2 | 2 | 0.1674 | 0.81 | essential |
| ko00780 | Biotin metabolism | 7 | 15 | 0.1749 | 0.83 | essential |
| ko00790 | Folate biosynthesis | 8 | 21 | 0.2129 | 0.97 | essential |
| ko00040 | Pentose and glucuronate interconversions | 1 | 17 | 0.2124 | 0.97 | nonessential |
| ko01120 | Microbial metabolism in diverse environments | 41 | 226 | 0.2199 | 0.98 | nonessential |
| ko04931 | Insulin resistance | 2 | 3 | 0.2274 | 0.99 | essential |
| ko00770 | Pantothenate and CoA biosynthesis | 8 | 21 | 0.2329 | 1.00 | essential |
| ko01110 | Biosynthesis of secondary metabolites | 76 | 295 | 0.3888 | 1.00 | essential |
| ko01130 | Biosynthesis of antibiotics | 53 | 217 | 0.6822 | 1.00 | essential |
| ko01200 | Carbon metabolism | 22 | 123 | 0.3378 | 1.00 | nonessential |
| ko01230 | Biosynthesis of amino acids | 26 | 127 | 0.6837 | 1.00 | nonessential |
| ko00230 | Purine metabolism | 19 | 74 | 0.6972 | 1.00 | essential |
| ko00260 | Glycine, serine and threonine metabolism | 5 | 40 | 0.2644 | 1.00 | nonessential |
| ko00720 | Carbon fixation pathways in prokaryotes | 12 | 36 | 0.2659 | 1.00 | essential |
| ko00520 | Amino sugar and nucleotide sugar metabolism | 7 | 36 | 0.8456 | 1.00 | nonessential |
| ko00650 | Butanoate metabolism | 5 | 35 | 0.4193 | 1.00 | nonessential |
| ko00640 | Propanoate metabolism | 6 | 30 | 0.8441 | 1.00 | nonessential |
| ko00270 | Cysteine and methionine metabolism | 7 | 35 | 0.8476 | 1.00 | nonessential |
| ko00250 | Alanine, aspartate and glutamate metabolism | 4 | 30 | 0.3793 | 1.00 | nonessential |
| ko00400 | Phenylalanine, tyrosine and tryptophan biosynthesis | 6 | 23 | 0.8206 | 1.00 | essential |
| ko00030 | Pentose phosphate pathway | 5 | 25 | 0.8141 | 1.00 | nonessential |
| ko01210 | 2-Oxocarboxylic acid metabolism | 5 | 26 | 0.8191 | 1.00 | nonessential |
| ko00564 | Glycerophospholipid metabolism | 6 | 18 | 0.4078 | 1.00 | essential |
| ko00710 | Carbon fixation in photosynthetic organisms | 4 | 25 | 0.6367 | 1.00 | nonessential |
| ko03018 | RNA degradation | 5 | 18 | 0.7866 | 1.00 | essential |
| ko00760 | Nicotinate and nicotinamide metabolism | 5 | 16 | 0.5632 | 1.00 | essential |
| ko01501 | beta-Lactam resistance | 4 | 17 | 1.0000 | 1.00 | essential |
| ko02026 | Biofilm formation - Escherichia coli | 3 | 20 | 0.5812 | 1.00 | nonessential |
| ko00670 | One carbon pool by folate | 4 | 17 | 1.0000 | 1.00 | essential |
| ko00910 | Nitrogen metabolism | 2 | 17 | 0.4048 | 1.00 | nonessential |
| ko03410 | Base excision repair | 4 | 15 | 1.0000 | 1.00 | essential |
| ko00480 | Glutathione metabolism | 3 | 19 | 0.6202 | 1.00 | nonessential |
| ko00310 | Lysine degradation | 2 | 18 | 0.4008 | 1.00 | nonessential |
| ko00362 | Benzoate degradation | 1 | 14 | 0.3178 | 1.00 | nonessential |
| ko00290 | Valine, leucine and isoleucine biosynthesis | 1 | 14 | 0.3383 | 1.00 | nonessential |
| ko00660 | C5-Branched dibasic acid metabolism | 2 | 11 | 1.0000 | 1.00 | nonessential |
| ko00071 | Fatty acid degradation | 1 | 15 | 0.3498 | 1.00 | nonessential |
| ko01503 | Cationic antimicrobial peptide | 2 | 12 | 0.7666 | 1.00 | nonessential |
| ko05111 | Biofilm formation - Vibrio cholerae | 2 | 13 | 0.7556 | 1.00 | nonessential |
| ko04146 | Peroxisome | 2 | 11 | 1.0000 | 1.00 | nonessential |
| ko00360 | Phenylalanine metabolism | 1 | 13 | 0.3423 | 1.00 | nonessential |
| ko04122 | Sulfur relay system | 2 | 12 | 0.7666 | 1.00 | nonessential |
| ko00561 | Glycerolipid metabolism | 2 | 12 | 0.7581 | 1.00 | nonessential |
| ko00380 | Tryptophan metabolism | 1 | 15 | 0.3173 | 1.00 | nonessential |
| ko03420 | Nucleotide excision repair | 2 | 8 | 1.0000 | 1.00 | essential |
| ko00521 | Streptomycin biosynthesis | 1 | 12 | 0.4838 | 1.00 | nonessential |
| ko00730 | Thiamine metabolism | 3 | 12 | 1.0000 | 1.00 | essential |
| ko01220 | Degradation of aromatic compounds | 3 | 9 | 0.7136 | 1.00 | essential |
| ko00562 | Inositol phosphate metabolism | 2 | 10 | 1.0000 | 1.00 | nonessential |
| ko00410 | beta-Alanine metabolism | 1 | 11 | 0.5022 | 1.00 | nonessential |
| ko00450 | Selenocompound metabolism | 3 | 11 | 1.0000 | 1.00 | essential |
| ko05016 | Huntington's disease | 4 | 8 | 0.2514 | 1.00 | essential |
| ko00983 | Drug metabolism - other enzymes | 1 | 7 | 1.0000 | 1.00 | nonessential |
| ko00430 | Taurine and hypotaurine metabolism | 0 | 8 | 0.3583 | 1.00 | nonessential |
| ko00750 | Vitamin B6 metabolism | 1 | 6 | 1.0000 | 1.00 | nonessential |
| ko04212 | Longevity regulating pathway - worm | 4 | 8 | 0.2524 | 1.00 | essential |
| ko00906 | Carotenoid biosynthesis | 0 | 5 | 0.5837 | 1.00 | nonessential |
| ko00072 | Synthesis and degradation of ketone bodies | 1 | 8 | 0.7116 | 1.00 | nonessential |
| ko05134 | Legionellosis | 2 | 10 | 1.0000 | 1.00 | nonessential |
| ko02060 | Phosphotransferase system | 1 | 5 | 1.0000 | 1.00 | nonessential |
| ko00523 | Polyketide sugar unit biosynthesis | 0 | 6 | 0.3578 | 1.00 | nonessential |
| ko00625 | Chloroalkane and chloroalkene degradation | 0 | 8 | 0.3718 | 1.00 | nonessential |
| ko00053 | Ascorbate and aldarate metabolism | 0 | 8 | 0.3553 | 1.00 | nonessential |
| ko01523 | Antifolate resistance | 3 | 6 | 0.3938 | 1.00 | essential |
| ko05010 | Alzheimer's disease | 3 | 9 | 0.7161 | 1.00 | essential |
| ko00261 | Monobactam biosynthesis | 4 | 8 | 0.2534 | 1.00 | essential |
| ko05152 | Tuberculosis | 2 | 9 | 1.0000 | 1.00 | nonessential |
| ko04066 | HIF-1 signaling pathway | 0 | 6 | 0.3753 | 1.00 | nonessential |
| ko04932 | Non-alcoholic fatty liver disease | 3 | 6 | 0.3778 | 1.00 | essential |
| ko04922 | Glucagon signaling pathway | 0 | 5 | 0.6037 | 1.00 | nonessential |
| ko00401 | Novobiocin biosynthesis | 0 | 6 | 0.3648 | 1.00 | nonessential |
| ko05012 | Parkinson's disease | 3 | 6 | 0.3768 | 1.00 | essential |
| ko00460 | Cyanoamino acid metabolism | 0 | 5 | 0.6052 | 1.00 | nonessential |
| ko05230 | Central carbon metabolism in cancer | 0 | 5 | 0.6012 | 1.00 | nonessential |
| ko00471 | D-Glutamine and D-glutamate metabolism | 2 | 4 | 0.6097 | 1.00 | essential |
| ko04213 | Longevity regulating pathway - multiple species | 1 | 5 | 1.0000 | 1.00 | nonessential |
| ko00960 | Tropane, piperidine and pyridine alkaloid biosynthesis | 0 | 5 | 0.5917 | 1.00 | nonessential |
| ko04070 | Phosphatidylinositol signaling system | 2 | 5 | 0.6162 | 1.00 | essential |
| ko00333 | Prodigiosin biosynthesis | 4 | 10 | 0.5002 | 1.00 | essential |
| ko04016 | MAPK signaling pathway - plant | 1 | 4 | 1.0000 | 1.00 | essential |
| ko05418 | Fluid shear stress and atherosclerosis | 1 | 8 | 0.6892 | 1.00 | nonessential |
| ko04217 | Necroptosis | 1 | 7 | 1.0000 | 1.00 | nonessential |
| ko01040 | Biosynthesis of unsaturated fatty acids | 1 | 8 | 0.7046 | 1.00 | nonessential |
| ko01524 | Platinum drug resistance | 1 | 10 | 0.5127 | 1.00 | nonessential |
| ko05014 | Amyotrophic lateral sclerosis | 0 | 6 | 0.3923 | 1.00 | nonessential |
| ko04626 | Plant-pathogen interaction | 1 | 4 | 1.0000 | 1.00 | essential |
| ko00791 | Atrazine degradation | 0 | 3 | 0.6397 | 1.00 | nonessential |
| ko00633 | Nitrotoluene degradation | 0 | 4 | 0.5987 | 1.00 | nonessential |
| ko00643 | Styrene degradation | 0 | 3 | 0.6192 | 1.00 | nonessential |
| ko00473 | D-Alanine metabolism | 1 | 4 | 1.0000 | 1.00 | essential |
| ko05204 | Chemical carcinogenesis | 1 | 7 | 1.0000 | 1.00 | nonessential |
| ko00281 | Geraniol degradation | 1 | 2 | 1.0000 | 1.00 | essential |
| ko00622 | Xylene degradation | 0 | 2 | 1.0000 | 1.00 | nonessential |
| ko00626 | Naphthalene degradation | 1 | 2 | 1.0000 | 1.00 | essential |
| ko04621 | NOD-like receptor signaling pathway | 0 | 3 | 0.6287 | 1.00 | nonessential |
| ko00405 | Phenazine biosynthesis | 0 | 2 | 1.0000 | 1.00 | nonessential |
| ko00950 | Isoquinoline alkaloid biosynthesis | 0 | 3 | 0.6257 | 1.00 | nonessential |
| ko04214 | Apoptosis - fly | 0 | 4 | 0.5862 | 1.00 | nonessential |
| ko04068 | FoxO signaling pathway | 1 | 3 | 1.0000 | 1.00 | essential |
| ko00525 | Acarbose and validamycin biosynthesis | 0 | 2 | 1.0000 | 1.00 | nonessential |
| ko00361 | Chlorocyclohexane and chlorobenzene degradation | 0 | 2 | 1.0000 | 1.00 | nonessential |
| ko04910 | Insulin signaling pathway | 0 | 3 | 0.6317 | 1.00 | nonessential |
| ko03320 | PPAR signaling pathway | 0 | 2 | 1.0000 | 1.00 | nonessential |
| ko00930 | Caprolactam degradation | 0 | 2 | 1.0000 | 1.00 | nonessential |
| ko00627 | Aminobenzoate degradation | 0 | 2 | 1.0000 | 1.00 | nonessential |
| ko05120 | Epithelial cell signaling in Helicobacter pylori infection | 0 | 2 | 1.0000 | 1.00 | nonessential |
| ko00903 | Limonene and pinene degradation | 0 | 5 | 0.6022 | 1.00 | nonessential |
| ko04918 | Thyroid hormone synthesis | 0 | 2 | 1.0000 | 1.00 | nonessential |
| ko05200 | Pathways in cancer | 1 | 4 | 1.0000 | 1.00 | essential |
| ko03450 | Non-homologous end-joining | 0 | 3 | 0.6337 | 1.00 | nonessential |
| ko00440 | Phosphonate and phosphinate metabolism | 0 | 2 | 1.0000 | 1.00 | nonessential |
| ko04211 | Longevity regulating pathway | 0 | 3 | 0.6387 | 1.00 | nonessential |
| ko00980 | Metabolism of xenobiotics by cytochrome P450 | 1 | 7 | 1.0000 | 1.00 | nonessential |
| ko05132 | Salmonella infection | 0 | 4 | 0.5857 | 1.00 | nonessential |
| ko00982 | Drug metabolism - cytochrome P450 | 1 | 7 | 1.0000 | 1.00 | nonessential |
| ko00940 | Phenylpropanoid biosynthesis | 0 | 2 | 1.0000 | 1.00 | nonessential |
| ko00332 | Carbapenem biosynthesis | 0 | 2 | 1.0000 | 1.00 | nonessential |
| ko01055 | Biosynthesis of vancomycin group antibiotics | 0 | 1 | 1.0000 | 1.00 | nonessential |
| ko00621 | Dioxin degradation | 1 | 1 | 0.3323 | 1.00 | essential |
| ko05219 | Bladder cancer | 0 | 1 | 1.0000 | 1.00 | nonessential |
| ko05206 | MicroRNAs in cancer | 0 | 1 | 1.0000 | 1.00 | nonessential |
| ko04215 | Apoptosis - multiple species | 0 | 3 | 0.6487 | 1.00 | nonessential |
| ko05167 | Kaposi's sarcoma-associated herpesvirus infection | 0 | 3 | 0.6392 | 1.00 | nonessential |
| ko05161 | Hepatitis B | 0 | 3 | 0.6397 | 1.00 | nonessential |
| ko05231 | Choline metabolism in cancer | 0 | 1 | 1.0000 | 1.00 | nonessential |
| ko04115 | p53 signaling pathway | 0 | 3 | 0.6367 | 1.00 | nonessential |
| ko05222 | Small cell lung cancer | 0 | 3 | 0.6312 | 1.00 | nonessential |
| ko05145 | Toxoplasmosis | 0 | 3 | 0.6287 | 1.00 | nonessential |
| ko05203 | Viral carcinogenesis | 0 | 2 | 1.0000 | 1.00 | nonessential |
| ko05205 | Proteoglycans in cancer | 0 | 1 | 1.0000 | 1.00 | nonessential |
| ko05210 | Colorectal cancer | 0 | 3 | 0.6332 | 1.00 | nonessential |
| ko04138 | Autophagy - yeast | 0 | 1 | 1.0000 | 1.00 | nonessential |
| ko05340 | Primary immunodeficiency | 0 | 1 | 1.0000 | 1.00 | nonessential |
| ko00472 | D-Arginine and D-ornithine metabolism | 0 | 2 | 1.0000 | 1.00 | nonessential |
| ko04013 | MAPK signaling pathway - fly | 1 | 1 | 0.3523 | 1.00 | essential |
| ko05164 | Influenza A | 0 | 3 | 0.6357 | 1.00 | nonessential |
| ko05146 | Amoebiasis | 0 | 1 | 1.0000 | 1.00 | nonessential |
| ko00311 | Penicillin and cephalosporin biosynthesis | 1 | 1 | 0.3273 | 1.00 | essential |
| ko04964 | Proximal tubule bicarbonate reclamation | 0 | 1 | 1.0000 | 1.00 | nonessential |
| ko00121 | Secondary bile acid biosynthesis | 0 | 1 | 1.0000 | 1.00 | nonessential |
| ko04216 | Ferroptosis | 0 | 1 | 1.0000 | 1.00 | nonessential |
| ko04978 | Mineral absorption | 0 | 1 | 1.0000 | 1.00 | nonessential |
| ko00965 | Betalain biosynthesis | 0 | 1 | 1.0000 | 1.00 | nonessential |
| ko04152 | AMPK signaling pathway | 0 | 2 | 1.0000 | 1.00 | nonessential |
| ko01051 | Biosynthesis of ansamycins | 1 | 2 | 1.0000 | 1.00 | essential |
| ko00524 | Neomycin, kanamycin and gentamicin biosynthesis | 0 | 1 | 1.0000 | 1.00 | nonessential |
| ko05211 | Renal cell carcinoma | 1 | 1 | 0.3513 | 1.00 | essential |
| ko00590 | Arachidonic acid metabolism | 0 | 1 | 1.0000 | 1.00 | nonessential |
| ko04080 | Neuroactive ligand-receptor interaction | 0 | 1 | 1.0000 | 1.00 | nonessential |
| ko05168 | Herpes simplex infection | 0 | 3 | 0.6357 | 1.00 | nonessential |
| ko05020 | Prion diseases | 0 | 1 | 1.0000 | 1.00 | nonessential |
| ko00981 | Insect hormone biosynthesis | 0 | 4 | 0.5802 | 1.00 | nonessential |
| ko05225 | Hepatocellular carcinoma | 1 | 6 | 1.0000 | 1.00 | nonessential |
| ko01053 | Biosynthesis of siderophore group nonribosomal peptides | 0 | 1 | 1.0000 | 1.00 | nonessential |
| ko00830 | Retinol metabolism | 0 | 1 | 1.0000 | 1.00 | nonessential |
| ko04930 | Type II diabetes mellitus | 0 | 2 | 1.0000 | 1.00 | nonessential |
| ko03008 | Ribosome biogenesis in eukaryotes | 0 | 1 | 1.0000 | 1.00 | nonessential |
| ko04940 | Type I diabetes mellitus | 1 | 3 | 1.0000 | 1.00 | essential |
| ko00624 | Polycyclic aromatic hydrocarbon degradation | 1 | 1 | 0.3183 | 1.00 | essential |
| ko05416 | Viral myocarditis | 0 | 3 | 0.6377 | 1.00 | nonessential |
| ko00120 | Primary bile acid biosynthesis | 0 | 1 | 1.0000 | 1.00 | nonessential |
| ko00600 | Sphingolipid metabolism | 0 | 1 | 1.0000 | 1.00 | nonessential |
| ko05166 | HTLV-I infection | 0 | 1 | 1.0000 | 1.00 | nonessential |
| ko04210 | Apoptosis | 0 | 3 | 0.6152 | 1.00 | nonessential |
| ko05150 | Staphylococcus aureus infection | 0 | 1 | 1.0000 | 1.00 | nonessential |
| ko00966 | Glucosinolate biosynthesis | 0 | 1 | 1.0000 | 1.00 | nonessential |
| ko05133 | Pertussis | 1 | 1 | 0.3303 | 1.00 | essential |
| ko04727 | GABAergic synapse | 1 | 5 | 1.0000 | 1.00 | nonessential |
| ko04011 | MAPK signaling pathway - yeast | 0 | 2 | 1.0000 | 1.00 | nonessential |
| ko00531 | Glycosaminoglycan degradation | 0 | 1 | 1.0000 | 1.00 | nonessential |
| ko04724 | Glutamatergic synapse | 1 | 5 | 1.0000 | 1.00 | nonessential |
| ko00908 | Zeatin biosynthesis | 1 | 1 | 0.3523 | 1.00 | essential |
| ko04920 | Adipocytokine signaling pathway | 0 | 1 | 1.0000 | 1.00 | nonessential |
| ko04072 | Phospholipase D signaling pathway | 0 | 1 | 1.0000 | 1.00 | nonessential |

**Table S5**: **Pathway essentiality predictions of *Rhizobium leguminosarum***

| KO | Pathway | Essential genes | All | p-value | fdr | Predicted essentiality |
| --- | --- | --- | --- | --- | --- | --- |
| ko00195 | Photosynthesis | 8 | 8 | 0.0005 | 0.01 | essential |
| ko00970 | Aminoacyl-tRNA biosynthesis | 17 | 25 | 0.0005 | 0.01 | essential |
| ko01120 | Microbial metabolism in diverse environments | 12 | 220 | 0.0005 | 0.01 | nonessential |
| ko03010 | Ribosome | 40 | 51 | 0.0005 | 0.01 | essential |
| ko02010 | ABC transporters | 8 | 179 | 0.0015 | 0.02 | nonessential |
| ko00540 | Lipopolysaccharide biosynthesis | 8 | 11 | 0.0025 | 0.02 | essential |
| ko00550 | Peptidoglycan biosynthesis | 11 | 16 | 0.0020 | 0.02 | essential |
| ko03030 | DNA replication | 10 | 15 | 0.0025 | 0.02 | essential |
| ko00061 | Fatty acid biosynthesis | 8 | 13 | 0.0055 | 0.03 | essential |
| ko00190 | Oxidative phosphorylation | 18 | 51 | 0.0050 | 0.03 | essential |
| ko04112 | Cell cycle - Caulobacter | 11 | 26 | 0.0050 | 0.03 | essential |
| ko03430 | Mismatch repair | 8 | 17 | 0.0130 | 0.07 | essential |
| ko01212 | Fatty acid metabolism | 8 | 18 | 0.0190 | 0.10 | essential |
| ko01502 | Vancomycin resistance | 4 | 5 | 0.0230 | 0.11 | essential |
| ko01110 | Biosynthesis of secondary metabolites | 23 | 238 | 0.0300 | 0.14 | nonessential |
| ko02020 | Two-component system | 4 | 71 | 0.0350 | 0.15 | nonessential |
| ko00630 | Glyoxylate and dicarboxylate metabolism | 1 | 36 | 0.0525 | 0.20 | nonessential |
| ko03020 | RNA polymerase | 3 | 4 | 0.0535 | 0.20 | essential |
| ko01130 | Biosynthesis of antibiotics | 17 | 174 | 0.0565 | 0.21 | nonessential |
| ko01230 | Biosynthesis of amino acids | 8 | 100 | 0.0670 | 0.23 | nonessential |
| ko03440 | Homologous recombination | 7 | 19 | 0.0825 | 0.27 | essential |
| ko00240 | Pyrimidine metabolism | 12 | 46 | 0.1164 | 0.32 | essential |
| ko00260 | Glycine, serine and threonine metabolism | 2 | 40 | 0.1054 | 0.32 | nonessential |
| ko00270 | Cysteine and methionine metabolism | 1 | 29 | 0.1164 | 0.32 | nonessential |
| ko00780 | Biotin metabolism | 3 | 6 | 0.1129 | 0.32 | essential |
| ko02024 | Quorum sensing | 4 | 58 | 0.1479 | 0.39 | nonessential |
| ko00860 | Porphyrin and chlorophyll metabolism | 2 | 35 | 0.1639 | 0.40 | nonessential |
| ko03060 | Protein export | 5 | 15 | 0.1634 | 0.40 | essential |
| ko01200 | Carbon metabolism | 9 | 93 | 0.1864 | 0.44 | nonessential |
| ko01100 | Metabolic pathways | 83 | 620 | 0.2064 | 0.46 | nonessential |
| ko05152 | Tuberculosis | 2 | 4 | 0.2014 | 0.46 | essential |
| ko00471 | D-Glutamine and D-glutamate metabolism | 2 | 5 | 0.2404 | 0.51 | essential |
| ko04212 | Longevity regulating pathway - worm | 2 | 5 | 0.2449 | 0.51 | essential |
| ko04940 | Type I diabetes mellitus | 1 | 1 | 0.2609 | 0.53 | essential |
| ko00220 | Arginine biosynthesis | 1 | 20 | 0.3478 | 0.65 | nonessential |
| ko00400 | Phenylalanine, tyrosine and tryptophan biosynthesis | 1 | 21 | 0.3463 | 0.65 | nonessential |
| ko00910 | Nitrogen metabolism | 1 | 18 | 0.3393 | 0.65 | nonessential |
| ko00230 | Purine metabolism | 14 | 68 | 0.4033 | 0.72 | essential |
| ko00900 | Terpenoid backbone biosynthesis | 3 | 10 | 0.4048 | 0.72 | essential |
| ko00730 | Thiamine metabolism | 3 | 11 | 0.4298 | 0.74 | essential |
| ko00620 | Pyruvate metabolism | 3 | 31 | 0.4668 | 0.79 | nonessential |
| ko00710 | Carbon fixation in photosynthetic organisms | 1 | 15 | 0.5027 | 0.81 | nonessential |
| ko03410 | Base excision repair | 1 | 14 | 0.5062 | 0.81 | nonessential |
| ko03018 | RNA degradation | 4 | 15 | 0.5272 | 0.83 | essential |
| ko00020 | Citrate cycle | 2 | 21 | 0.5737 | 0.88 | nonessential |
| ko00450 | Selenocompound metabolism | 2 | 8 | 0.6427 | 0.92 | essential |
| ko00640 | Propanoate metabolism | 3 | 30 | 0.6292 | 0.92 | nonessential |
| ko04122 | Sulfur relay system | 2 | 8 | 0.6372 | 0.92 | essential |
| ko00520 | Amino sugar and nucleotide sugar metabolism | 4 | 33 | 0.6587 | 0.93 | nonessential |
| ko00561 | Glycerolipid metabolism | 1 | 12 | 0.7021 | 0.97 | nonessential |
| ko00030 | Pentose phosphate pathway | 4 | 26 | 1.0000 | 1.00 | nonessential |
| ko00130 | Ubiquinone and other terpenoid-quinone biosynthesis | 1 | 9 | 1.0000 | 1.00 | nonessential |
| ko00300 | Lysine biosynthesis | 2 | 12 | 1.0000 | 1.00 | essential |
| ko00333 | Prodigiosin biosynthesis | 1 | 3 | 1.0000 | 1.00 | essential |
| ko00473 | D-Alanine metabolism | 1 | 3 | 1.0000 | 1.00 | essential |
| ko00480 | Glutathione metabolism | 2 | 13 | 1.0000 | 1.00 | nonessential |
| ko00564 | Glycerophospholipid metabolism | 3 | 20 | 1.0000 | 1.00 | nonessential |
| ko00720 | Carbon fixation pathways in prokaryotes | 5 | 25 | 0.7781 | 1.00 | essential |
| ko00740 | Riboflavin metabolism | 1 | 7 | 1.0000 | 1.00 | nonessential |
| ko00750 | Vitamin B metabolism | 1 | 7 | 1.0000 | 1.00 | nonessential |
| ko01210 | -Oxocarboxylic acid metabolism | 3 | 21 | 1.0000 | 1.00 | nonessential |
| ko01501 | beta-Lactam resistance | 2 | 13 | 1.0000 | 1.00 | nonessential |
| ko01503 | Cationic antimicrobial peptide | 1 | 7 | 1.0000 | 1.00 | nonessential |
| ko03070 | Bacterial secretion system | 4 | 26 | 1.0000 | 1.00 | nonessential |
| ko03420 | Nucleotide excision repair | 1 | 8 | 1.0000 | 1.00 | nonessential |
| ko04070 | Phosphatidylinositol signaling system | 1 | 3 | 1.0000 | 1.00 | essential |
| ko04146 | Peroxisome | 1 | 7 | 1.0000 | 1.00 | nonessential |
| ko05134 | Legionellosis | 1 | 6 | 1.0000 | 1.00 | essential |
| ko05230 | Central carbon metabolism in cancer | 1 | 6 | 1.0000 | 1.00 | essential |

**Table S6: Pathway essentiality predictions of *Caulobacter crescentus***

| KO | Pathway | Essential genes | All | p-value | fdr | Predicted essentiality |
| --- | --- | --- | --- | --- | --- | --- |
| ko02020 | Two-component system | 9 | 115 | 0.0005 | 0.03 | nonessential |
| ko03010 | Ribosome | 49 | 54 | 0.0005 | 0.03 | essential |
| ko00970 | Aminoacyl-tRNA biosynthesis | 22 | 24 | 0.0005 | 0.03 | essential |
| ko00540 | Lipopolysaccharide biosynthesis | 13 | 14 | 0.0005 | 0.03 | essential |
| ko00190 | Oxidative phosphorylation | 29 | 50 | 0.0010 | 0.04 | essential |
| ko02030 | Bacterial chemotaxis | 0 | 42 | 0.0015 | 0.06 | nonessential |
| ko02040 | Flagellar assembly | 0 | 34 | 0.0045 | 0.14 | nonessential |
| ko00195 | Photosynthesis | 9 | 9 | 0.0055 | 0.15 | essential |
| ko01100 | Metabolic pathways | 189 | 601 | 0.0080 | 0.18 | essential |
| ko03060 | Protein export | 12 | 17 | 0.0080 | 0.18 | essential |
| ko00280 | Valine, leucine and isoleucine degradation | 2 | 42 | 0.0105 | 0.21 | nonessential |
| ko03030 | DNA replication | 11 | 16 | 0.0130 | 0.24 | essential |
| ko00550 | Peptidoglycan biosynthesis | 12 | 19 | 0.0160 | 0.26 | essential |
| ko00362 | Benzoate degradation | 0 | 24 | 0.0160 | 0.26 | nonessential |
| ko00061 | Fatty acid biosynthesis | 12 | 18 | 0.0180 | 0.26 | essential |
| ko00730 | Thiamine metabolism | 8 | 10 | 0.0185 | 0.26 | essential |
| ko00240 | Pyrimidine metabolism | 20 | 43 | 0.0230 | 0.30 | essential |
| ko00020 | Citrate cycle | 13 | 22 | 0.0280 | 0.35 | essential |
| ko03430 | Mismatch repair | 10 | 17 | 0.0320 | 0.38 | essential |
| ko00630 | Glyoxylate and dicarboxylate metabolism | 4 | 45 | 0.0445 | 0.50 | nonessential |
| ko00340 | Histidine metabolism | 0 | 20 | 0.0470 | 0.50 | nonessential |
| ko04112 | Cell cycle - Caulobacter | 15 | 32 | 0.0585 | 0.60 | essential |
| ko00300 | Lysine biosynthesis | 8 | 14 | 0.0660 | 0.62 | essential |
| ko00071 | Fatty acid degradation | 1 | 24 | 0.0660 | 0.62 | nonessential |
| ko00720 | Carbon fixation pathways in prokaryotes | 15 | 34 | 0.0755 | 0.63 | essential |
| ko00360 | Phenylalanine metabolism | 1 | 22 | 0.0710 | 0.63 | nonessential |
| ko01524 | Platinum drug resistance | 0 | 14 | 0.0740 | 0.63 | nonessential |
| ko00380 | Tryptophan metabolism | 1 | 23 | 0.0790 | 0.63 | nonessential |
| ko00900 | Terpenoid backbone biosynthesis | 9 | 18 | 0.0880 | 0.68 | essential |
| ko02010 | ABC transporters | 8 | 61 | 0.0950 | 0.69 | nonessential |
| ko00350 | Tyrosine metabolism | 0 | 15 | 0.0950 | 0.69 | nonessential |
| ko01502 | Vancomycin resistance | 4 | 6 | 0.1144 | 0.78 | essential |
| ko00460 | Cyanoamino acid metabolism | 0 | 11 | 0.1154 | 0.78 | nonessential |
| ko00860 | Porphyrin and chlorophyll metabolism | 9 | 19 | 0.1384 | 0.82 | essential |
| ko05111 | Biofilm formation - Vibrio cholerae | 1 | 18 | 0.1364 | 0.82 | nonessential |
| ko00750 | Vitamin B6 metabolism | 4 | 6 | 0.1294 | 0.82 | essential |
| ko04626 | Plant-pathogen interaction | 0 | 12 | 0.1399 | 0.82 | nonessential |
| ko00404 | Staurosporine biosynthesis | 0 | 11 | 0.1259 | 0.82 | nonessential |
| ko00910 | Nitrogen metabolism | 1 | 17 | 0.1479 | 0.83 | nonessential |
| ko03020 | RNA polymerase | 3 | 4 | 0.1454 | 0.83 | essential |
| ko03440 | Homologous recombination | 9 | 20 | 0.1564 | 0.83 | essential |
| ko00680 | Methane metabolism | 1 | 17 | 0.1519 | 0.83 | nonessential |
| ko00920 | Sulfur metabolism | 1 | 19 | 0.1609 | 0.83 | nonessential |
| ko00710 | Carbon fixation in photosynthetic organisms | 7 | 14 | 0.1629 | 0.83 | essential |
| ko00010 | Glycolysis / Gluconeogenesis | 3 | 29 | 0.1859 | 0.85 | nonessential |
| ko00650 | Butanoate metabolism | 5 | 38 | 0.1994 | 0.85 | nonessential |
| ko00330 | Arginine and proline metabolism | 3 | 26 | 0.2399 | 0.85 | nonessential |
| ko00770 | Pantothenate and CoA biosynthesis | 8 | 18 | 0.2199 | 0.85 | essential |
| ko01220 | Degradation of aromatic compounds | 1 | 15 | 0.2329 | 0.85 | nonessential |
| ko00290 | Valine, leucine and isoleucine biosynthesis | 1 | 15 | 0.2249 | 0.85 | nonessential |
| ko00130 | Ubiquinone and other terpenoid-quinone biosynthesis | 5 | 10 | 0.2054 | 0.85 | essential |
| ko00564 | Glycerophospholipid metabolism | 6 | 13 | 0.2324 | 0.85 | essential |
| ko00310 | Lysine degradation | 2 | 21 | 0.2079 | 0.85 | nonessential |
| ko05016 | Huntington's disease | 0 | 9 | 0.2149 | 0.85 | nonessential |
| ko00627 | Aminobenzoate degradation | 0 | 10 | 0.2389 | 0.85 | nonessential |
| ko00053 | Ascorbate and aldarate metabolism | 0 | 8 | 0.2109 | 0.85 | nonessential |
| ko00930 | Caprolactam degradation | 0 | 10 | 0.2299 | 0.85 | nonessential |
| ko00072 | Synthesis and degradation of ketone bodies | 1 | 15 | 0.2099 | 0.85 | nonessential |
| ko00903 | Limonene and pinene degradation | 0 | 8 | 0.2339 | 0.85 | nonessential |
| ko00940 | Phenylpropanoid biosynthesis | 0 | 9 | 0.2179 | 0.85 | nonessential |
| ko00982 | Drug metabolism - cytochrome P450 | 1 | 15 | 0.2169 | 0.85 | nonessential |
| ko04621 | NOD-like receptor signaling pathway | 0 | 8 | 0.2194 | 0.85 | nonessential |
| ko00785 | Lipoic acid metabolism | 2 | 2 | 0.1799 | 0.85 | essential |
| ko01110 | Biosynthesis of secondary metabolites | 73 | 250 | 0.2534 | 0.89 | essential |
| ko00780 | Biotin metabolism | 6 | 14 | 0.2579 | 0.89 | essential |
| ko04070 | Phosphatidylinositol signaling system | 2 | 3 | 0.2684 | 0.91 | essential |
| ko00270 | Cysteine and methionine metabolism | 4 | 30 | 0.2794 | 0.93 | nonessential |
| ko00260 | Glycine, serine and threonine metabolism | 4 | 30 | 0.2869 | 0.94 | nonessential |
| ko00500 | Starch and sucrose metabolism | 2 | 20 | 0.2889 | 0.94 | nonessential |
| ko00051 | Fructose and mannose metabolism | 1 | 13 | 0.3148 | 0.96 | nonessential |
| ko00521 | Streptomycin biosynthesis | 1 | 12 | 0.3223 | 0.96 | nonessential |
| ko00410 | beta-Alanine metabolism | 1 | 14 | 0.3218 | 0.96 | nonessential |
| ko05230 | Central carbon metabolism in cancer | 0 | 6 | 0.3663 | 0.96 | nonessential |
| ko05010 | Alzheimer's disease | 0 | 7 | 0.3443 | 0.96 | nonessential |
| ko00625 | Chloroalkane and chloroalkene degradation | 0 | 6 | 0.3663 | 0.96 | nonessential |
| ko04932 | Non-alcoholic fatty liver disease | 0 | 6 | 0.3593 | 0.96 | nonessential |
| ko05012 | Parkinson's disease | 0 | 6 | 0.3623 | 0.96 | nonessential |
| ko04212 | Longevity regulating pathway - worm | 3 | 5 | 0.3743 | 0.96 | essential |
| ko05418 | Fluid shear stress and atherosclerosis | 1 | 13 | 0.3333 | 0.96 | nonessential |
| ko00361 | Chlorocyclohexane and chlorobenzene degradation | 0 | 6 | 0.3738 | 0.96 | nonessential |
| ko00623 | Toluene degradation | 0 | 6 | 0.3573 | 0.96 | nonessential |
| ko00980 | Metabolism of xenobiotics by cytochrome P450 | 1 | 13 | 0.3303 | 0.96 | nonessential |
| ko05204 | Chemical carcinogenesis | 1 | 13 | 0.3218 | 0.96 | nonessential |
| ko05211 | Renal cell carcinoma | 1 | 1 | 0.3458 | 0.96 | essential |
| ko05132 | Salmonella infection | 0 | 7 | 0.3823 | 0.96 | nonessential |
| ko04940 | Type I diabetes mellitus | 1 | 1 | 0.3623 | 0.96 | essential |
| ko01051 | Biosynthesis of ansamycins | 1 | 1 | 0.3788 | 0.96 | essential |
| ko04016 | MAPK signaling pathway - plant | 1 | 1 | 0.3733 | 0.96 | essential |
| ko00908 | Zeatin biosynthesis | 1 | 1 | 0.3788 | 0.96 | essential |
| ko00333 | Prodigiosin biosynthesis | 3 | 6 | 0.3978 | 0.99 | essential |
| ko00040 | Pentose and glucuronate interconversions | 2 | 16 | 0.4058 | 1.00 | nonessential |
| ko01120 | Microbial metabolism in diverse environments | 42 | 192 | 0.4943 | 1.00 | nonessential |
| ko01130 | Biosynthesis of antibiotics | 55 | 210 | 0.7451 | 1.00 | essential |
| ko01230 | Biosynthesis of amino acids | 26 | 107 | 0.9115 | 1.00 | nonessential |
| ko01200 | Carbon metabolism | 26 | 110 | 0.8456 | 1.00 | nonessential |
| ko00230 | Purine metabolism | 17 | 60 | 0.6592 | 1.00 | essential |
| ko03070 | Bacterial secretion system | 11 | 33 | 0.4323 | 1.00 | essential |
| ko00520 | Amino sugar and nucleotide sugar metabolism | 10 | 42 | 1.0000 | 1.00 | nonessential |
| ko00620 | Pyruvate metabolism | 9 | 37 | 1.0000 | 1.00 | nonessential |
| ko02024 | Quorum sensing | 8 | 27 | 0.6777 | 1.00 | essential |
| ko00250 | Alanine, aspartate and glutamate metabolism | 4 | 26 | 0.4938 | 1.00 | nonessential |
| ko00790 | Folate biosynthesis | 5 | 22 | 1.0000 | 1.00 | nonessential |
| ko01210 | 2-Oxocarboxylic acid metabolism | 5 | 27 | 0.6517 | 1.00 | nonessential |
| ko00400 | Phenylalanine, tyrosine and tryptophan biosynthesis | 6 | 22 | 1.0000 | 1.00 | essential |
| ko00030 | Pentose phosphate pathway | 7 | 20 | 0.4833 | 1.00 | essential |
| ko01212 | Fatty acid metabolism | 12 | 36 | 0.4563 | 1.00 | essential |
| ko00640 | Propanoate metabolism | 7 | 33 | 0.8461 | 1.00 | nonessential |
| ko03018 | RNA degradation | 5 | 15 | 0.5687 | 1.00 | essential |
| ko00220 | Arginine biosynthesis | 3 | 17 | 0.7906 | 1.00 | nonessential |
| ko00760 | Nicotinate and nicotinamide metabolism | 5 | 15 | 0.5787 | 1.00 | essential |
| ko00670 | One carbon pool by folate | 2 | 13 | 0.7621 | 1.00 | nonessential |
| ko00480 | Glutathione metabolism | 4 | 26 | 0.5012 | 1.00 | nonessential |
| ko03410 | Base excision repair | 4 | 15 | 1.0000 | 1.00 | essential |
| ko01501 | beta-Lactam resistance | 2 | 17 | 0.4248 | 1.00 | nonessential |
| ko00052 | Galactose metabolism | 2 | 15 | 0.5377 | 1.00 | nonessential |
| ko00562 | Inositol phosphate metabolism | 2 | 13 | 0.7576 | 1.00 | nonessential |
| ko01503 | Cationic antimicrobial peptide | 2 | 13 | 0.7381 | 1.00 | nonessential |
| ko00450 | Selenocompound metabolism | 3 | 10 | 1.0000 | 1.00 | essential |
| ko00660 | C5-Branched dibasic acid metabolism | 2 | 10 | 1.0000 | 1.00 | nonessential |
| ko04122 | Sulfur relay system | 4 | 9 | 0.4653 | 1.00 | essential |
| ko03420 | Nucleotide excision repair | 1 | 8 | 0.7051 | 1.00 | nonessential |
| ko00740 | Riboflavin metabolism | 1 | 8 | 0.7026 | 1.00 | nonessential |
| ko00561 | Glycerolipid metabolism | 3 | 8 | 0.7126 | 1.00 | essential |
| ko04146 | Peroxisome | 2 | 7 | 1.0000 | 1.00 | essential |
| ko00261 | Monobactam biosynthesis | 3 | 7 | 0.4338 | 1.00 | essential |
| ko05134 | Legionellosis | 2 | 14 | 0.5412 | 1.00 | nonessential |
| ko02026 | Biofilm formation - Escherichia coli | 1 | 8 | 0.6987 | 1.00 | nonessential |
| ko01040 | Biosynthesis of unsaturated fatty acids | 1 | 9 | 0.6827 | 1.00 | nonessential |
| ko01523 | Antifolate resistance | 2 | 5 | 0.6177 | 1.00 | essential |
| ko00622 | Xylene degradation | 0 | 4 | 0.6092 | 1.00 | nonessential |
| ko04066 | HIF-1 signaling pathway | 0 | 4 | 0.5917 | 1.00 | nonessential |
| ko00471 | D-Glutamine and D-glutamate metabolism | 2 | 4 | 0.6077 | 1.00 | essential |
| ko00983 | Drug metabolism - other enzymes | 0 | 4 | 0.5972 | 1.00 | nonessential |
| ko00523 | Polyketide sugar unit biosynthesis | 0 | 5 | 0.5927 | 1.00 | nonessential |
| ko05152 | Tuberculosis | 2 | 6 | 1.0000 | 1.00 | essential |
| ko00511 | Other glycan degradation | 0 | 4 | 0.5822 | 1.00 | nonessential |
| ko00281 | Geraniol degradation | 1 | 7 | 0.7131 | 1.00 | nonessential |
| ko04142 | Lysosome | 0 | 4 | 0.5872 | 1.00 | nonessential |
| ko04922 | Glucagon signaling pathway | 0 | 4 | 0.5927 | 1.00 | nonessential |
| ko02060 | Phosphotransferase system | 0 | 5 | 0.5807 | 1.00 | nonessential |
| ko00430 | Taurine and hypotaurine metabolism | 0 | 5 | 0.5822 | 1.00 | nonessential |
| ko00401 | Novobiocin biosynthesis | 0 | 5 | 0.5687 | 1.00 | nonessential |
| ko00791 | Atrazine degradation | 0 | 3 | 0.6132 | 1.00 | nonessential |
| ko00531 | Glycosaminoglycan degradation | 0 | 3 | 0.6307 | 1.00 | nonessential |
| ko00643 | Styrene degradation | 0 | 4 | 0.5847 | 1.00 | nonessential |
| ko00473 | D-Alanine metabolism | 1 | 3 | 1.0000 | 1.00 | essential |
| ko02025 | Biofilm formation - Pseudomonas aeruginosa | 0 | 3 | 0.6167 | 1.00 | nonessential |
| ko04213 | Longevity regulating pathway - multiple species | 0 | 4 | 0.5967 | 1.00 | nonessential |
| ko04260 | Cardiac muscle contraction | 0 | 3 | 0.6142 | 1.00 | nonessential |
| ko00332 | Carbapenem biosynthesis | 0 | 2 | 1.0000 | 1.00 | nonessential |
| ko05206 | MicroRNAs in cancer | 0 | 3 | 0.6147 | 1.00 | nonessential |
| ko04217 | Necroptosis | 1 | 5 | 1.0000 | 1.00 | nonessential |
| ko04918 | Thyroid hormone synthesis | 0 | 2 | 1.0000 | 1.00 | nonessential |
| ko00960 | Tropane, piperidine and pyridine alkaloid biosynthesis | 0 | 4 | 0.5662 | 1.00 | nonessential |
| ko04724 | Glutamatergic synapse | 1 | 4 | 1.0000 | 1.00 | essential |
| ko00621 | Dioxin degradation | 0 | 2 | 1.0000 | 1.00 | nonessential |
| ko03320 | PPAR signaling pathway | 0 | 3 | 0.6077 | 1.00 | nonessential |
| ko00525 | Acarbose and validamycin biosynthesis | 0 | 2 | 1.0000 | 1.00 | nonessential |
| ko04214 | Apoptosis - fly | 0 | 4 | 0.6047 | 1.00 | nonessential |
| ko04210 | Apoptosis | 0 | 4 | 0.5962 | 1.00 | nonessential |
| ko00405 | Phenazine biosynthesis | 0 | 2 | 1.0000 | 1.00 | nonessential |
| ko05014 | Amyotrophic lateral sclerosis | 0 | 4 | 0.5912 | 1.00 | nonessential |
| ko00624 | Polycyclic aromatic hydrocarbon degradation | 0 | 2 | 1.0000 | 1.00 | nonessential |
| ko04727 | GABAergic synapse | 1 | 4 | 1.0000 | 1.00 | essential |
| ko00950 | Isoquinoline alkaloid biosynthesis | 0 | 3 | 0.6337 | 1.00 | nonessential |
| ko00440 | Phosphonate and phosphinate metabolism | 0 | 2 | 1.0000 | 1.00 | nonessential |
| ko05200 | Pathways in cancer | 1 | 4 | 1.0000 | 1.00 | essential |
| ko05120 | Epithelial cell signaling in Helicobacter pylori infection | 0 | 1 | 1.0000 | 1.00 | nonessential |
| ko05030 | Cocaine addiction | 0 | 2 | 1.0000 | 1.00 | nonessential |
| ko00590 | Arachidonic acid metabolism | 0 | 1 | 1.0000 | 1.00 | nonessential |
| ko00513 | Various types of N-glycan biosynthesis | 0 | 1 | 1.0000 | 1.00 | nonessential |
| ko05416 | Viral myocarditis | 0 | 3 | 0.6287 | 1.00 | nonessential |
| ko04931 | Insulin resistance | 1 | 3 | 1.0000 | 1.00 | essential |
| ko00966 | Glucosinolate biosynthesis | 0 | 1 | 1.0000 | 1.00 | nonessential |
| ko05150 | Staphylococcus aureus infection | 0 | 1 | 1.0000 | 1.00 | nonessential |
| ko04726 | Serotonergic synapse | 0 | 2 | 1.0000 | 1.00 | nonessential |
| ko04211 | Longevity regulating pathway | 0 | 2 | 1.0000 | 1.00 | nonessential |
| ko05145 | Toxoplasmosis | 0 | 3 | 0.6297 | 1.00 | nonessential |
| ko01055 | Biosynthesis of vancomycin group antibiotics | 0 | 1 | 1.0000 | 1.00 | nonessential |
| ko01053 | Biosynthesis of siderophore group nonribosomal peptides | 0 | 1 | 1.0000 | 1.00 | nonessential |
| ko00565 | Ether lipid metabolism | 0 | 1 | 1.0000 | 1.00 | nonessential |
| ko00524 | Neomycin, kanamycin and gentamicin biosynthesis | 0 | 2 | 1.0000 | 1.00 | nonessential |
| ko04964 | Proximal tubule bicarbonate reclamation | 0 | 1 | 1.0000 | 1.00 | nonessential |
| ko05142 | Chagas disease | 0 | 1 | 1.0000 | 1.00 | nonessential |
| ko03013 | RNA transport | 0 | 1 | 1.0000 | 1.00 | nonessential |
| ko00120 | Primary bile acid biosynthesis | 0 | 1 | 1.0000 | 1.00 | nonessential |
| ko05143 | African trypanosomiasis | 0 | 1 | 1.0000 | 1.00 | nonessential |
| ko05020 | Prion diseases | 0 | 1 | 1.0000 | 1.00 | nonessential |
| ko04068 | FoxO signaling pathway | 0 | 2 | 1.0000 | 1.00 | nonessential |
| ko05133 | Pertussis | 0 | 2 | 1.0000 | 1.00 | nonessential |
| ko05225 | Hepatocellular carcinoma | 1 | 11 | 0.4783 | 1.00 | nonessential |
| ko00830 | Retinol metabolism | 0 | 2 | 1.0000 | 1.00 | nonessential |
| ko05161 | Hepatitis B | 0 | 3 | 0.6167 | 1.00 | nonessential |
| ko04910 | Insulin signaling pathway | 0 | 1 | 1.0000 | 1.00 | nonessential |
| ko04215 | Apoptosis - multiple species | 0 | 3 | 0.6237 | 1.00 | nonessential |
| ko04614 | Renin-angiotensin system | 0 | 2 | 1.0000 | 1.00 | nonessential |
| ko00965 | Betalain biosynthesis | 0 | 1 | 1.0000 | 1.00 | nonessential |
| ko00626 | Naphthalene degradation | 0 | 2 | 1.0000 | 1.00 | nonessential |
| ko04930 | Type II diabetes mellitus | 0 | 1 | 1.0000 | 1.00 | nonessential |
| ko05205 | Proteoglycans in cancer | 0 | 1 | 1.0000 | 1.00 | nonessential |
| ko00364 | Fluorobenzoate degradation | 0 | 4 | 0.5902 | 1.00 | nonessential |
| ko00472 | D-Arginine and D-ornithine metabolism | 0 | 1 | 1.0000 | 1.00 | nonessential |
| ko04013 | MAPK signaling pathway - fly | 0 | 2 | 1.0000 | 1.00 | nonessential |
| ko04138 | Autophagy - yeast | 0 | 1 | 1.0000 | 1.00 | nonessential |
| ko04728 | Dopaminergic synapse | 0 | 2 | 1.0000 | 1.00 | nonessential |
| ko00604 | Glycosphingolipid biosynthesis - ganglio series | 0 | 1 | 1.0000 | 1.00 | nonessential |
| ko05164 | Influenza A | 0 | 3 | 0.6252 | 1.00 | nonessential |
| ko05034 | Alcoholism | 0 | 2 | 1.0000 | 1.00 | nonessential |
| ko01054 | Nonribosomal peptide structures | 0 | 2 | 1.0000 | 1.00 | nonessential |
| ko00600 | Sphingolipid metabolism | 0 | 1 | 1.0000 | 1.00 | nonessential |
| ko04974 | Protein digestion and absorption | 0 | 1 | 1.0000 | 1.00 | nonessential |
| ko05210 | Colorectal cancer | 0 | 3 | 0.5982 | 1.00 | nonessential |
| ko05031 | Amphetamine addiction | 0 | 2 | 1.0000 | 1.00 | nonessential |
| ko04919 | Thyroid hormone signaling pathway | 0 | 1 | 1.0000 | 1.00 | nonessential |
| ko05165 | Human papillomavirus infection | 0 | 1 | 1.0000 | 1.00 | nonessential |
| ko03008 | Ribosome biogenesis in eukaryotes | 0 | 1 | 1.0000 | 1.00 | nonessential |
| ko00981 | Insect hormone biosynthesis | 0 | 2 | 1.0000 | 1.00 | nonessential |
| ko05222 | Small cell lung cancer | 0 | 3 | 0.6107 | 1.00 | nonessential |
| ko05168 | Herpes simplex infection | 0 | 3 | 0.6232 | 1.00 | nonessential |
| ko00603 | Glycosphingolipid biosynthesis - globo and isoglobo series | 0 | 1 | 1.0000 | 1.00 | nonessential |
| ko00906 | Carotenoid biosynthesis | 0 | 1 | 1.0000 | 1.00 | nonessential |
| ko04115 | p53 signaling pathway | 0 | 3 | 0.6152 | 1.00 | nonessential |
| ko04152 | AMPK signaling pathway | 0 | 1 | 1.0000 | 1.00 | nonessential |
| ko05203 | Viral carcinogenesis | 0 | 1 | 1.0000 | 1.00 | nonessential |

**Figure S1: Transposon delivery vector pJG714 and amplification strategy for deep sequencing of transposon insertion sites
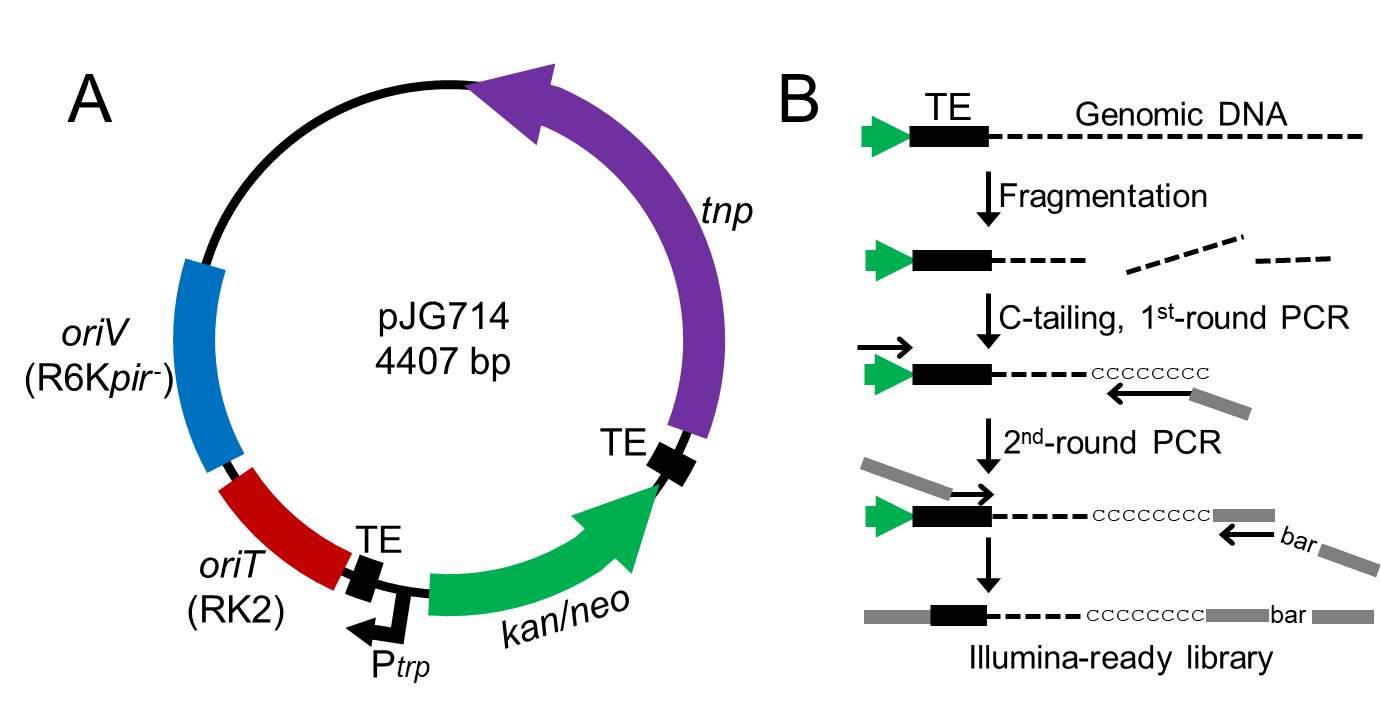
**
