# Supplementary material for: A Metagenome-Wide Association Study and Arrayed Mutant Library Confirm Acetobacter Lipopolysaccharide Genes Are Necessary for Association with Drosophila melanogaster
Source: G3 (Bethesda). 2018 Feb 27;8(4):1119–27. doi: 10.1534/g3.117.300530 (PMC5873903; doi:10.1534/g3.117.300530)
Supplement: Supplementary file 2 [file 1119FileS2.docx]

**File S2: Library preparation protocol.**

- Genomic DNA extraction using the DNeasy PowerLyzer Microbial DNA kit (Cat# 122550-50). Centrifuge 1.0-1.5 ml of overnight culture for two minutes. Remove supernatant and freeze pellet at -80°C for at least 30 minutes. Suspend pellet in 300 μl of MicroBead solution and transfer to bead tube. Add 50 μl buffer MD1; vortex for 10 minutes using bead beating adapter at full speed. Centrifuge for 3 minutes and move the supernatant to a new tube. Add 100 μl buffer MD2 and vortex for 5 second. Place on ice for 5 minutes before centrifuging for 3 minutes at full speed. Move supernatant (about 350 μl) to a new tube and add 900 μl buffer MD3. Mix using pipettor and then put through the spin filter 610 μl at a time, discarding flow through. Wash the spin filter with 300 μl buffer MD4, discarding flow through. Wash spin filter with 500 μl buffer PE from a Qiagen kit, discard flowthrough. Centrifuge for 1 minute at full speed and then move filter to a new tube. Add 55 μl warm 2.5 mm Tris (pH 8.0). Wait 2 minutes before centrifuging for 1 minute at full speed. The sample can be checked by running 2 μl on a gel and should be a bright high molecular-weight band. Check that the sample is at least 150ng/μl on nanodrop and normalize all samples to the lowest concentration.
- Fragementase digestions (NEB M0348S): Mix 16 ul of genomic DNA extract with 2 μl 10X fragmentase v2 buffer. Pre-vortex Fragmentase before adding 2 μl to the mixture and mixing immediately. Incubate at 37°C for exactly 12 minutes (time will vary, optimize time for each batch of Fragmentase). Add 10 μl 0.25 M EDTA to stop the reaction and keep on ice until clean up.
- Zymo DNA Clean and Concentrator - 25: Add 100 μl DNA Binding Buffer to the fragmentase reaction product. Transfer to column and centrifuge for 30 seconds. Discard flow through. Add 200 μl DNA Wash Buffer and centrifuge for 30 seconds and then repeat the wash step. To elute, transfer column to a new tube and add 50 μl warm 2.5 mM Tris (pH 8.0), wait two minutes and centrifuge for 1 minute at full speed. Check 4 μl on a gel for a smear from about 500-3000 bp.
- C tailing (TdT is NEB M0315S, ddCTP is Affimetrix 77112 0.5UM): Each reaction includes 30 μl cleaned up fragmented DNA, 4 μl 10 TdT buffer, 4 μl 2.5 mM CoC1_2_, 2.1 μl 9.5 mM dCTP/0.5mMddCTP mix (make mix with 34 μl water, 4 μl 100 mM dCTP 2 μl 10mMddCTP), 0.6 μl TdT enzyme. Incubate for 30 minutes at 37°C.
- Zymo clean up: Same as above
- First round PCR: Each reaction includes 17.8 μl water, 8.0 μl 5X Q5 buffer, 1.2 μl 10mM dNTPs, 0.5 μl Q5 polymerase, 2.5 μl 10mM 1 TN, 5.0 μl 10μM primer 1OLIGOG, 5 μl template DNA from cleaned up TdT reaction. *(Q5 is NEB M0491S)*
- Zymo clean up: Same as above.
- Second round PCR: 22.0 μl water, 8.0 5X Q5 buffer, 0.5 μl Q5 polymerase, 3.0 μl 10 mM assigned 2TN primer 3.0 μl 10 μM assigned 2BAR primer, 2.5 μl cleaned up product from first round.
- Zymo clean up: Same as above but elute in T_5_E_0.5_.
- Gel: Check 4 μl on a gel. Should have 150-700 bp with a strong peak around 400 bp.
