# Supplementary material for: A Metagenome-Wide Association Study and Arrayed Mutant Library Confirm Acetobacter Lipopolysaccharide Genes Are Necessary for Association with Drosophila melanogaster
Source: G3 (Bethesda). 2018 Feb 27;8(4):1119–27. doi: 10.1534/g3.117.300530 (PMC5873903; doi:10.1534/g3.117.300530)
Supplement: Supplementary file 8 [file 1119FileS8.docx]

**File S8: *Acetobacter* Conjugation Trials**

Liquid cultures of *E. coli* and *Acetobacter* were prepared in LB broth and YPd broth (10% yeast extract, 10% peptone, 8% dextrose), respectively. Cultures were incubated shaking at 30°C for 20 hours. *E. coli* WM 2655 (Larson et al., 2002) was used as a donor for pRL27 carrying a kanamycin marked mini-Tn5 transposon, and was cultivated with 100 ug/ml kanamycin. *E. coli* S17-1(λpir) was used as a donor for pCM62 (Marx & Lidstrom, 2001), a broad-host-range plasmid that can replicate in *Acetobacter* (Chaston et al., 2015), and was cultivated with 10 ug/ml tetracycline. Each trial was conducted at least two times, and representative data are presented.

Following cultivation, *Acetobacter* strains were normalized to an optical density of 0.2/ml, approx. 1.5 x 10^7^ CFU/ml. One ml of *Acetobacter* cells, and 250 ul of *E. coli* were subsequently pelleted and washed in fresh medium twice, and finally resuspended in 150 ul of YPd broth. Cell suspensions were mixed in a 1:1 volumetric ratio, providing roughly equal cell numbers of donor and recipient, then spotted in 50ul aliquots onto YPd agar. Each *Acetobacter* strain was also incubated alone as a negative control. Plates were incubated for 3 hours at 30°C. Next, cells were collected from conjugation and negative control plates in 1 ml of fresh YPd broth using a sterile spreader, generating a uniform suspension. Resuspended cells were plated onto selective media (kamamycin 100 ug/ml, 0.2% acetic acid; *or* tetracycline 20 ug/ml, 0.2% acetic acid) at various dilutions, and incubated for 3 days at 30°C.

*A. fabarum* DsW_054 showed the highest conjugation efficiency (14% = # of exconjugants/ # starting recipients) with the mini-Tn*5* vector, and thus was chosen for library construction. It also had a high conjugation efficiency with the tetracycline-marked replicating plasmid pCM62 (23%). Other strains that showed relatively high efficiencies with both donors included *A. tropicalis* DmW_042 and *Acetobacter* sp. DsW_059 (Table S2). Of the strains tested, only one did not yield exconjugants from either conjugation: *A. malorum* DsW_057. Four *Acetobacter* strains showed a high rate of spontaneous resistance to kanamycin (Table S2), and the following strains were not included in the analysis due to significant background resistance to tetracycline and kanamycin: *A. orientalis* DmW_045; *A. orientalis* DmW_048; *A. indonesiensis* DmL_051; *A. okinawensis* DsW_060; *A. nitrogenifigens* DsW_063.

**Works cited**
Chaston JM, Newell PD, Douglas AE. 2014. Metagenome-wide association of microbial determinants of host phenotype in Drosophila melanogaster. *mBio* 5(5):e01631-14.

Larsen RA, Wilson MM, Guss AM, Metcalf WW. 2002. Genetic analysis of pigment biosynthesis in *Xanthobacter autotrophicus* Py2 using a new, highly efficient transposon mutagenesis system that is functional in a wide variety of bacteria. *Arch Microbiol.* 178(3):193-201.

Marx CJ, Lidstrom ME. 2001. Development of improved versatile broad-host-range vectors for use in methylotrophs and other gram-negative bacteria. *Microbiol.* 147(8):2065–2075

Winans NJ, Walter A, Chouaia B, Chaston JM, Douglas AE, Newell PD. 2017. A genomic investigation of ecological differentiation between free-living and Drosophila-associated bacteria. *Mol Ecol.* 26(17):4536-4550.
